# The RNA binding proteins TIA1 and TIAL1 promote *Mcl1* mRNA translation to protect germinal center responses from apoptosis

**DOI:** 10.1038/s41423-023-01063-4

**Published:** 2023-07-20

**Authors:** Ines C. Osma-Garcia, Mailys Mouysset, Dunja Capitan-Sobrino, Yann Aubert, Martin Turner, Manuel D. Diaz-Muñoz

**Affiliations:** 1 Toulouse Institute for Infectious and Inflammatory Diseases (INFINITy), Inserm UMR1291, CNRS UMR5051, University Paul Sabatier, CHU Purpan, Toulouse, 31024 France; 2 Immunology Program, The Babraham Institute, Cambridge, UK

**Keywords:** Adaptive immunity, Germinal centers, Post-transcriptional gene regulation, RNA binding proteins, Cell identity, Apoptosis, Germinal centres, Gene regulation in immune cells, Immune cell death

## Abstract

Germinal centers (GCs) are essential for the establishment of long-lasting antibody responses. GC B cells rely on post-transcriptional RNA mechanisms to translate activation-associated transcriptional programs into functional changes in the cell proteome. However, the critical proteins driving these key mechanisms are still unknown. Here, we show that the RNA binding proteins TIA1 and TIAL1 are required for the generation of long-lasting GC responses. TIA1- and TIAL1-deficient GC B cells fail to undergo antigen-mediated positive selection, expansion and differentiation into B-cell clones producing high-affinity antibodies. Mechanistically, TIA1 and TIAL1 control the transcriptional identity of dark- and light-zone GC B cells and enable timely expression of the prosurvival molecule MCL1. Thus, we demonstrate here that TIA1 and TIAL1 are key players in the post-transcriptional program that selects high-affinity antigen-specific GC B cells.

## Introduction

Germinal centers (GCs) are the sites in secondary lymph organs where B cells undergo affinity maturation and differentiation into long-lasting memory B cells and high-affinity antibody-producing plasma cells. Affinity maturation of GC B cells relies on the fine coordination of antigen recognition through the B-cell receptor (BCR) with activation, proliferation, somatic hypermutation (SHM) and selection of B-cell clones [1, 2]. Gene expression programs controlling each of these processes are tightly modulated in GC B cells, both at the transcriptional and post-transcriptional levels [3–5].

Gene transcription is closely linked with the functional regulation of nascent RNA through interactions with RNA binding proteins (RBPs). RBPs control the splicing, editing, translation and decay of mRNAs involved in the establishment, expansion and differentiation of GC cells [6]. Genetic ablation of key RBPs, such as HuR and PTBP1, has a direct impact on the expression of master transcription factors (TFs) and TF-associated gene signatures in GC B cells, highlighting the importance of post-transcriptional mechanisms in GC-mediated immune responses [7–9]. HuR, PTBP1 and IGF2BP3 directly regulate the expression of MYC and the MYC-associated gene signature that mediates antigen-dependent selection and expansion of GC B cells [10, 11]. In addition, these molecules secure cell energy fueling and DNA replication and contribute to the mitigation of extensive DNA damage introduced by activation-induced deaminase (AID) in rapidly proliferating GC B cells. More recently, a genetic screen targeting RBPs identified dozens of these proteins having a relevant role in the terminal differentiation of GC B cells into memory and plasma cells [12]. This included members of the RNA deadenylation complex and decay machinery that are central for this terminal differentiation process, and they act coordinately in the maintenance of long-lived plasma cells [13, 14].

Multiple other RBPs are believed to contribute to launching and maintaining GC-mediated immunity, as they are as abundant as TFs. However, to date, only a handful of RBPs have been shown to be relevant for the GC reaction. Identification of the RNA targets of these key RBPs often reveals dozens of functionally related mRNAs that are commonly deregulated in knockout (KO) GC B cells [15]. Similarly, the same mRNA molecule can be bound by several proteins. Taken together, these findings suggest that RBPs must constitute networks for post-transcriptional gene regulation in GC B cells [16].

Here, we identify TIA1 and TIA-like 1 (TIAL1) as essential RBPs for the maintenance of long-lasting GC responses and the production of high-affinity class-switched antibodies. TIA1 and TIAL1 were first described in cancer-infiltrating cytotoxic T cells [17–19]. However, their physiological relevance in lymphocytes remains unclear. TIA1 and TIAL1 contain three RNA recognition motifs (RRMs) that recognize U-rich elements present in the introns and 3' untranslated region (3'UTRs) of their target mRNAs and are involved mostly in the regulation of mRNA splicing and translation. The association of TIA1 and TIAL1 with their RNA targets is often coupled with cell detection of internal and external cues, allowing timely expression of key mRNA targets for cell growth and proliferation (e.g., *Myc*, *Gadd45a* and *Trp53*) [20–22]. TIA1 and TIAL1 deletion is embryonic lethal in mice [23, 24]. They are needed for the repair of physiological DNA damage in developing B cells [25] and control p53 mRNA translation after mature B-cell activation with mitogens [26]. Here, we reveal the intrinsic role of TIA1 and TIAL1 in GC B cells, show that these RBPs are essential for the generation of high-affinity antibody responses, and identify *Mcl1* as one of the key mRNA targets of TIA1 and TIAL1 that allows the selection and survival of antigen-specific GC B cells.

## Materials and methods

### Animal models

Knockout-first *Tia1*^*tm1a (KOMP)Wtsi*^ and *Tia1*^*tm1a(EUCOMM)Wtsi*^ mice, generated by the Welcome-Trust Sanger Institute as part of the International Knockout Mouse Consortium (IKMC), including the European Conditional Mouse Mutagenesis Program (EUCOMM, www.eucomm.org; and the Knock Out Mouse Project (KOMP, www.komp.org), were crossed with Tg(*Aicda-cre*)9^Mbu^ (*AID*^*Cre*^) mice [27] to generate single *Tia1 (Tia1*^*fl/fl*^ *AID*^*Cre*^*)*, single *Tial1* (*Tial1*^*fl/fl*^ *AID*^*Cre*^) or double *Tia1 Tial1* (*Tia1*^*fl/fl*^ *Tial1*^*fl/fl*^ *AID*^*Cre*^) conditional KO (cKO) mice. C57BL/6-Ly5.1 (CD45.1^+^) and *Rag2*^*−/−*^ mice were obtained from The Jackson Laboratory (Bar Harbor, US). All mice were maintained on a C57BL/6 background. Randomization, but not experimental ‘blinding’, was set in these studies by housing cKO mice and Cre-negative littermate control mice in the same cage from weaning. Male and female mice were used indistinctively at an age of 8–16 weeks old. No primary pathogens or additional agents listed in the FELASA recommendations were confirmed during health monitoring surveys of the mouse stock. The ambient temperature was ~19–21 °C, and the relative humidity was 52%. Lighting was provided on a 12-hour light: 12-hour dark cycle. After weaning, mice were transferred to individually ventilated cages with 2–5 mice per cage. Food was provided ad libitum, and cages received environmental enrichment. All experimental procedures were approved by the local ethical committee of INFINITy and by the French Ministry of Education, Research and Innovation.

### Animal procedures

Immune responses were induced upon intraperitoneal (ip) or subcutaneous (sc) injection of 100 μg. or 25 μg. of 4-hydroxy-3-nitrophenylacetyl hapten conjugated to Keyhole Limpet Hemocyanin (NP-KLH, Biosearch Technologies) precipitated in aluminum hydroxide gel (Serva). No adjuvant was used in NP-KLH recall responses. For in vivo analysis of the cell cycle, animals were injected with 2 mg. of BrdU (Sigma Aldrich) in 200 µl of PBS ip and culled 1.5 hours after injection.

Bone marrow chimera mice were generated as previously described [8]. Briefly, bone marrow cells were isolated from the tibias and femurs of C57BL/6-Ly5.1 (CD45.1^+^) mice and *Tia1*^*fl/fl*^ *Tial1*^*fl/fl*^ and *Tia1*^*fl/fl*^ *Tial1*^*fl/fl*^ *AID*^*Cre*^ mice (both CD45.2^+^), treated with ACK lysis buffer (Thermo Scientific), counted and mixed at a 1:4 ratio (CD45.1/CD45.2). A total of 5 × 10^6^ cells were i.v. injected into sublethally irradiated *Rag2*^*−/−*^ mice (X-ray irradiation, 500 Gy). Immunization was performed 8 weeks after BM reconstitution.

### B-cell isolation and cell culture

Follicular (FO) B cells were isolated from spleens or peripheral lymph nodes using the B Cell Isolation Kit from Miltenyi Biotec. In vitro-derived GC B cells were generated as previously described [28]. Briefly, 17.5 × 10^3^ FO B cells and irradiated (120 Gy) 40LB stromal cells were cocultured in RPMI-1640 medium (Dutch Modification, Thermo Fisher Scientific) plus 10% FCS (Sigma-Aldrich)﻿, antibiotics (Sigma-Aldrich), 2 mM L-glutamine (Thermo Fisher Scientific), 1 mM sodium pyruvate (Thermo Fisher Scientific) and β-mercaptoethanol (50 μM, Thermo Fisher Scientific) in the presence of IL-4 (2 ng/ml, Peprotech). The medium was replaced every two days, and at day 4, iGC B cells were reseeded on freshly irradiated 40LB stromal cells. Analysis of cell differentiation into iGC B cells and class switching was analyzed by flow cytometry as indicated below. HEK 293 T cells from the ECACC collection were cultured on DMEM with Glutamax (Thermo Fisher Scientific), 10% FCS, antibiotics, 2 mM L-glutamine and 1 mM sodium pyruvate.

### Flow cytometry

GC B-cell responses were mainly assessed by flow cytometry using the antibodies listed in Supplemental Table 3. B-cell subsets were identified as follows: nonactivated B cells - CD19^+^ IgD^+^; GC B cells - CD19^+^ IgD^-^ CD38^-^ CD95^+^; DZ GC B cells - CD19^+^ IgD^-^ CD38^-^ CD95^+^ CXCR4^+^ CD86^-^; LZ GC B cells - CD19^+^ IgD^-^ CD38^-^ CD95^+^ CXCR4^-^ CD86^+^; and memory B cells CD19^+^ IgD^-^ NP^+^ CD38^+^ CD95^-^. Then, 4-hydroxy-3-nitrophenylacetyl hapten (NP, LGC Biosearch Technologies) conjugated to PE was used to detect antigen-specific B cells. Class-switched and MYC^+^ GC B cells were identified using specific antibodies.

Prior to cell surface staining with fluorescent antibodies, cells were incubated with an Fc receptor blocking antibody (clone 2.4G2) and with Zombie NIR Fixable Viability (BioLegend) for 15 min at 4 °C in PBS + 2% FCS (FACS buffer). After extensive washes, cells were incubated with the cell surface antibody mix for 30 min at 4 °C in FACS buffer. After washes with FACS buffer, staining of intracellular proteins was performed using BD Cytofix/Cytoperm™ Fixation and Permeabilization Solution from BD Biosciences. By default, this staining was performed O/N at 4 °C. BrdU and DNA staining was carried out using the FITC BrdU Flow Kit from BD Biosciences. Cell viability was assessed by combining Zombie NIR Fixable Viability and CaspACE™ FITC-VAD-FMK in Situ Marker from Promega (1 µM per 10^7^ cells incubated at 37 °C in RPMI-1640 containing 10% FCS, antibiotics, 10 mM HEPES, 2 mM L-glutamine, 1 mM sodium pyruvate and 50 μM β-mercaptoethanol for 30 minutes). For phospho-flow analyses, cells were fixed by dropwise addition of cold methanol followed by 4% PFA fixation and staining of surface and intracellular proteins. Data were collected in a BD Fortessa and analyzed with FlowJo vX.

### ELISA and ELISPOT assays

Total (NP14-) and high affinity (NP2-) antibodies were detected by ELISAs as previously described [29]. NP-specific antibody endpoint titers were used as a measure of relative concentration. NP-specific antibody secreting cells (ASCs) were detected by ELISpot as described [30]. The antibodies used are described in Supplemental Table 3.

### Confocal microscopy

The spleens of *Tia1*^*fl/fl*^ *Tial1*^*fl/fl*^ and *Tia1*^*fl/fl*^ *Tial1*^*fl/fl*^ *AID*^*Cre*^ mice immunized with NP-KLH in alum were embedded in Tissue-Tek O.C.T. (Sakura Finetek) before snap freezing and cryopreservation. Ten μm sections were fixed with acetone at -20 °C for 15 min. Then, at room temperature, slides were dried and blocked with PBS with 0.3% Triton X-100 and 5% BSA for 1 h. Staining with specific antibodies against IgD (coupled to AF488, 1 in 400), Ki67 (coupled to AF647, 1 in 100) and CD21/35 (coupled to biotin, 1 in 400) was performed O/N at 4 °C in a wet chamber. After washing, tissue samples were incubated with streptavidin-PE (1 in 800) for 45 min at room temperature. Sections were then washed (3x) with PBS with 0.3% Triton X100, dried and mounted using ProLong Gold Antifade Mounting (Thermo Scientific). Images were collected with a Leica SP8 confocal microscope using Leica Application Suite X (LAS X) software and analyzed with FIJI ImageJ.

### Plasmid constructs

Generation of MIGR1 plasmids containing a mouse wild-type TIA1, TIAL1 or a mutant TIA1 lacking the RNA recognition motifs 1 and 2 were described previously [26]. The dual-luciferase psiCheck2 reporter containing the mouse *Mcl1* 3′UTR (NCBI Reference Sequence: NM_008562.3) was generated by PCR amplification using B-cell mRNA as template, Pfu Ultra II Fusion HS polymerase (Thermo Fisher Scientific) and specific forward and reverse primers (CCTTGTGAGTGCAATAGGGGAC and TTGGGGGGAAAAAGGTTTATTTTCTCTTC). A hanging tail was added with the restriction enzyme sites of XhoI and NotI for directional cloning downstream of the Renilla luciferase ORF in the psiCheck2 plasmid. Deletion of the three major TIA1 and TIAL1 binding sites in the *Mcl1* 3′UTR was performed by Gibson cloning using the following primers: backbone forward CAGTAATTCTAGGCGATCGCTCGAGCCTTGTGAGTGCAATAG, backbone reverse GATATTTTATTGCGGCCAGCGGCCGCTTGGGGGGAAAA, *Mcl1* 3′UTR delta (d) 1 reverse AGTCAACAGGCTTTTTGGCCATCACTAGGC, *Mcl1* 3′UTR delta (d) 1 forward GGCCAAAAAGCCTGTTGACTTACAAAATTGTAAATGGTAAAGCAG, *Mcl1* 3′UTR delta (d) 2 reverse GCAAACTAATCAATGGAAAGCATGCCAATC, *Mcl1* 3′UTR delta (d) 2 forward CTTTCCATTGATTAGTTTGCTCCAAGTATGACTGTGTTCAC, *Mcl1* 3′UTR delta (d) 3 reverse AGAGTTAAACACTCCCAAGCTGGCAGGC, *Mcl1* 3′UTR delta (d) 3 forward GCTTGGGAGTGTTTAACTCTTCGGACTTCAGAGCAC.

### Dicer-substrate short interfering RNAs

TriFECTa Dicer substrate short interfering RNAs (DsiRNAs) against TIA1, TIAL1 and negative control were purchased from Integrated DNA Technologies (IDT, references: hs.Ri.TIA1.13.2, hs.Ri.TIAL1.13.2 and Negative Control DsiRNA). DsiRNAs (10 nM) were transfected into HEK293 cells using Lipofectamine RNAiMAX transfection reagent (Thermo Fisher). After 48 h, dual luciferase reporter plasmids and dsiRNA were cotransfected into the cells by using Lipofectamine 2000 (Thermo Fisher). Gene knockdown was evaluated 24 h after cotransfection by RT‒qPCR.

### Luciferase reporter assays

psiCheck2 and MIGR1 plasmids were cotransfected into HEK293T cells using Lipofectamine 2000 (Thermo Fisher Scientific) as previously described [26]. Renilla and firefly luciferase expression was measured after 48 h using a Dual-Luciferase® Reporter Assay System (Promega) as indicated by the manufacturer. At least three independent experiments were performed in triplicate. Data are shown as relative luciferase units calculated by dividing Renilla luciferase units by Firefly luciferase units. Then, values are relatively quantified to the control value. Pooled data are shown in each of the figure panels. Mann‒Whitney tests were performed for statistical analyses.

### Cell sorting and RNA sequencing

DZ and LZ GC B cells from *Tia1*^*fl/fl*^ *Tial1*^*fl/fl*^ and *Tia1*^*fl/fl*^ *Tial1*^*fl/fl*^ *AID*^*Cre*^ mice immunized with NP-KLH alum were FACS-sorted 7 days post-immunization as previously described [8]. Briefly, GC B cells were pre-enriched by depleting IgD^+^, CD3e^+^, Gr1^+^ and Ter119^+^ cells with biotinylated antibodies (Supplemental Table 3), anti-biotin magnetic beads and MACS LS columns (Miltenyi Biotec). GC B cells were then labeled as follows: LZ GC B cells - CD19^+^ CD38^-^ CD95^+^ GL7^+^ CXCR4^-^ CD86^+^; DZ GC B cells - CD19^+^ CD38^-^ CD95^+^ GL7^+^ CXCR4^+^ CD86^-^. Cells were FACS-sorted using a BD FACSAria Fusion sorter into complete medium. RNA was isolated using the RNeasy Micro Kit from Qiagen, and libraries were generated using the NEBNext® Single Cell/Low Input RNA Library Prep Kit for Illumina® (New England Biolabs [NEB], Cat No. E6420L). NEBNext® Multiplex Oligos for Illumina® (Dual Index Primers Set 1 or 2, NEB, Cat No. E7780S) were used for library production and dual indexing of samples. Libraries were multiplexed and sequenced by BGI (China) in four lanes of a DNBSeq platform (100 bp paired-end mode).

### Individual crosslinking immunoprecipitation (iCLIP)

Individual nucleotide crosslinking immunoprecipitation (iCLIP) [31, 32] was used to identify the RNA interactome of TIA1 and TIAL1 in B cells. Briefly, follicular B cells were isolated from the spleen and LNs of C57BL/6 mice. A total of 30×10^6^ cells were stimulated at 37 °C in RPMI-1640 containing 10% FCS, antibiotics, 10 mM HEPES, 2 mM L-glutamine, 1 mM sodium pyruvate and 50 μM β-mercaptoethanol with 5 µg/ml αIgM (F(ab’)_2_ fragment, Jackson ImmunoResearch), 10 µg/ml αCD40 (clone FGK4.5, Bioxcell) and 10 ng/ml rmIL-4 (Peprotech). After 72 h, the cells were washed with ice-cold PBS and irradiated with UV light (600 mJ/cm2) using a Stratalinker 2400. Cell lysis was performed with RIPA buffer and sonication (10 s, x3) for clarification. After centrifugation at 15000 rpm, samples were treated with TurboDNAse (Thermo Fisher Scientific), and RNA was partially digested with RNase I (0.167 U/ml, Thermo Fisher) for 3 minutes at 37 °C. Then, 3 μg of αTIA1 (clone EPR11323(B), Abcam) or αTIAL1 (clone D32D2, Cell Signaling Technologies) antibody was coupled to protein G Dynabeads (Thermo Fisher) for 1 h at RT prior to adding total cell extracts for immunoprecipitation. A rabbit IgG isotype control (clone DA1E, Cell Signaling Technologies) was used as a negative control. After extensive washes of immunoprecipitants with high-salt buffer (50 mM Tris-HCl pH 7.4, 1 M NaCl, 1 mM EDTA, 1% NP-40, 0.1% SDS and 0.5% sodium deoxycholate) and with PNK wash buffer (20 mM Tris-HCl pH 7.4, 10 mM MgCl2, 0.2% Tween-20), RNA was 3' end dephosphorylated using the enzymes FastAP alkaline phosphatase (Thermo Fisher Scientific) and PNK (NEB) in PNK buffer (20 mM Tris-HCl pH 7.4, 10 mM MgCl2, 0.2% Tween-20). After washing, one tenth of the sample was ligated to a preadenylated infrared labeled L3-IR-App adapter [33] (/5rApp/AG ATC GGA AGA GCG GTT CAG AAA AAA AAA AAA /iAzideN/AA AAA AAA AAA A/3Bio/ coupled with IRdye-800CW-DBCO (LI-COR)) using T4 RNA ligase I (NEB) and PNK. The rest of the sample was ligated to a nonlabeled L3-ATT-App DNA Linker (/5rApp/WN ATT AGA TCG GAA GAG CGG TTC AG/3Bio/) for library preparation. RNA‒protein complexes were separated by SDS‒PAGE electrophoresis, transferred to a nitrocellulose membrane and visualized in a LI-COR Odyssey system. RNA extraction from the nitrocellulose membrane was performed with proteinase K in PK buffer (100 mM Tris-Cl pH 7.5, 100 mM NaCl, 1 mM EDTA and 0.2% SDS) at 50 °C for 60 minutes. RNA was isolated by phenol/chloroform extraction and ethanol precipitation. RNA was reverse transcribed into cDNA using SuperScript IV reverse transcriptase (Thermo Fisher Scientific) and irCLIP_ddRT_19 primer (/5Phos/WWW AATAC NNNN AGA TCG GAA GAG CGT CGT GAT/iSp18/GGA TCC/iSp18/TAC TGA ACC GC) or irCLIP_ddRT_13 primer (/5Phos/WWW TCCGG NNNN AGA TCG GAA GAG CGT CGT GAT/iSp18/GGA TCC/iSp18/TAC TGA ACC GC). After cDNA purification with Agencourt AMPure XP beads (Beckman Coulter), cDNA was circularized with CircLigase II (Epicentre), amplified by PCR using Solexa P5/P7 primers and sequenced in the Illumina HiSeq platform in the Genopole Toulouse (GenoToul, 50 bp, SE).

### Bioinformatics

For transcriptomics analyses, raw sequencing files were processed using the nextflow nf-core/RNAseq pipeline (v3.8.1). Reads from different sequencing lanes were concatenated using bash cat utility and trimmed with Cutadapt (v3.4) and Trimgalore (v.0.6.7). The quality of the resulting reads was assessed with FASTQC-0.11.9 using default parameters. Then, paired-end reads were aligned to the mouse genome (GRCm39-v106) and quantified with Salmon v1.5.2. Alignment files were indexed using SamTools (v. 1.15.1). R (v4.0.2) and DESeq2 (v1.28.1) [34] were used for differential expression analysis using default parameters. Conditions included genotype, cell type and sample preparation day to control for variation in the data due to these parameters. Changes in gene expression with a p value adjusted using Benjamini and Hochberg correction (padj)<0.05 were considered significant. iCLIP analyses were performed using iMaps (https://imaps.goodwright.com/) [26, 32]. Transcription factor activities were calculated using decoupleR 2.5.0 and the CollecTRI network [35]. Gene Ontology enrichment analyses were performed using Webgestalt [36] using default settings.

### Statistical analysis

Statistical analyses were performed in R (v. 4.0.2) or using Prism-GraphPad (v. 7.0.). Statistical tests are indicated in each figure legend. Student’s t tests or nonparametric Mann‒Whitney tests were used for comparisons between two groups (if not stated otherwise). The Kolmogorov–Smirnov test was used to assess changes in the empirical distribution function of two samples. The Benjamini and Hochberg test was used for multiple testing and false discovery rate calculation.

## Results

### The expression of TIA1, but not TIAL1, is increased in germinal centers

**Fig. 1:**
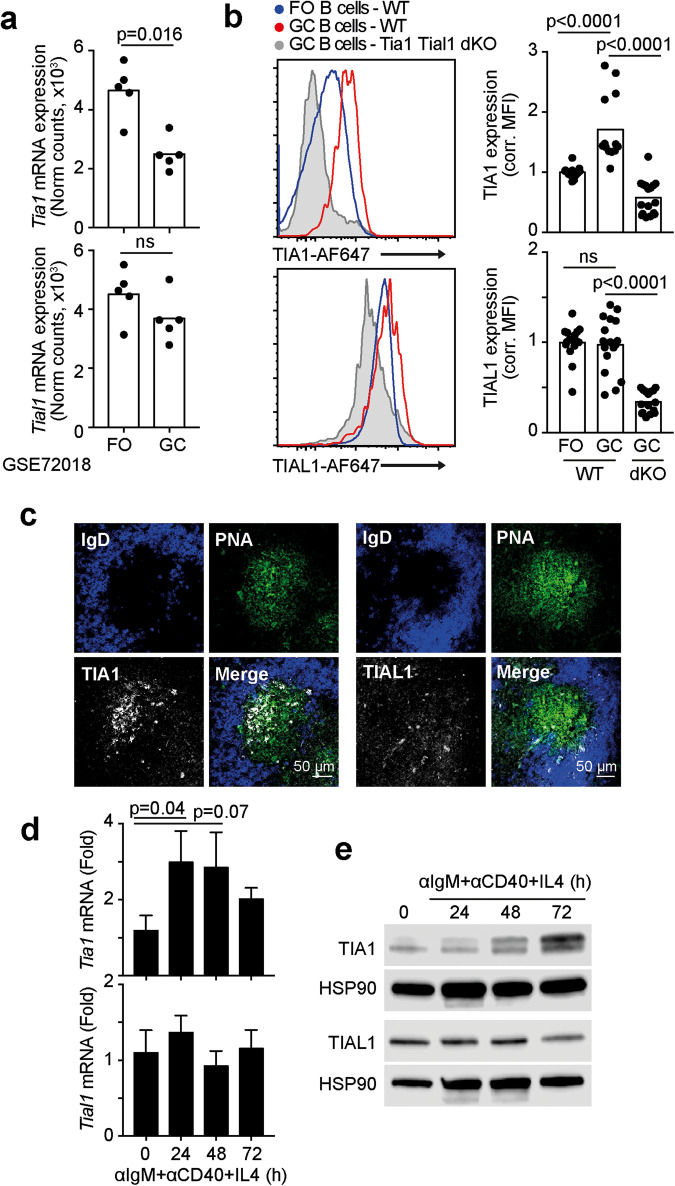
TIA1 expression is induced in antigen-activated GC B cells. **a** Expression of *Tia1* and *Tial1* mRNA in follicular (FO) and GC B cells. Transcriptomics data from dataset GSE72018 [68]. **b** Flow cytometry analysis of TIA1 and TIAL1 in FO and GC B cells. Left panels, representative histograms showing the expression of TIA1 and TIAL1. Right panels, quantitation of mean fluorescence intensity (MFI) corrected by the background signal from an isotype control antibody. Data from two independent experiments. Four and 5 mice were analyzed in each experiment. GC B cells from *Tia1*^*fl/fl*^ *Tial1*^*fl/fl*^ *AID*^*Cre*^ mice were used to assess antibody specificity. **c** Confocal imaging of TIA1 and TIAL1 expression in GCs induced with NP-KLH in alum (Day 7, 20x objective, IgD and PNA mark for naïve B cells and GC cells, respectively). **d** RT‒qPCR analysis of *Tia1* and *Tial1* mRNA abundance in B cells stimulated with anti-IgM, anti-CD40 and rmIL4 (n = 4 mice, mean ± SD). **e** Immunoblotting showing TIA1 and TIAL1 expression in B cells activated as in d. Mann‒Whitney tests were performed in (**b**) and (**d**)

To uncover the importance of the RBPs TIA1 and TIAL1 in adaptive immunity, we first investigated changes in the expression of these proteins in activated B cells. Transcriptomic studies consistently showed a significant reduction in *Tia1* mRNA expression in GC B cells compared to nonactivated follicular (FO) B cells, whereas the *Tial1* mRNA abundance remained constant (Fig. 1a). Indeed, flow cytometry analyses confirmed the absence of changes in TIAL1 protein expression between FO and GC B cells (Fig. 1b). However, TIA1 expression was found to be significantly increased in GC B cells (Fig. 1b). This discrepancy between mRNA and protein levels likely reflects the impact of post-transcriptional regulation on TIA1 expression, as previously suggested [37]. To confirm this observation, we performed in situ analyses of TIA1 and TIAL1 expression in spleen sections collected from mice immunized with the model antigen NP-KLH in alum (Fig. 1c). In these sections, TIA1 expression was mainly circumscribed to the GC area, with a lesser signal being detected in the IgD^+^ B-cell-containing follicle. Conversely, the TIAL1 signal was homogeneously distributed within the B-cell follicle. TIAL1 expression remained constant upon B-cell activation. In contrast, BCR-mediated stimulation significantly increased the expression of TIA1 at both the mRNA and protein levels (Fig. 1d and e). The kinetics of induction differed significantly, indicating a disconnection between RNA and protein levels. While the increase in RNA synthesis peaked at 24 hours and decreased later, a major amount of protein was found in B cells treated for 72 hours. Altogether, our results show that TIA1, but not TIAL1, is actively modulated in B cells and might have an important role in the GC reaction.

### Antibody responses are impaired in the absence of TIA1 and TIAL1

To study the intrinsic role of TIA1 and TIAL1 in GC cells, we generated single and double conditional knockout (KO) mice by crossing *Tia1*^*fl/fl*^ and *Tial1*^*fl/fl*^ mice with *Aicda-Cre* mice (hereafter named *AID*^*Cre*^ mice). Comparative analysis of TIA1 and TIAL1 expression in GC B cells from the control (*Tia1*^*fl/fl*^ *Tial1*^*fl/fl*^ Cre-negative littermates) and *Tia1*^*fl/fl*^ *Tial1*^*fl/fl*^ *AID*^*Cre*^ mice showed efficient gene deletion from the establishment of GCs at Day 3.5 post-immunization (Supplementary Fig. 1a, b), in agreement with our previous reports [8].

**Fig. 2:**
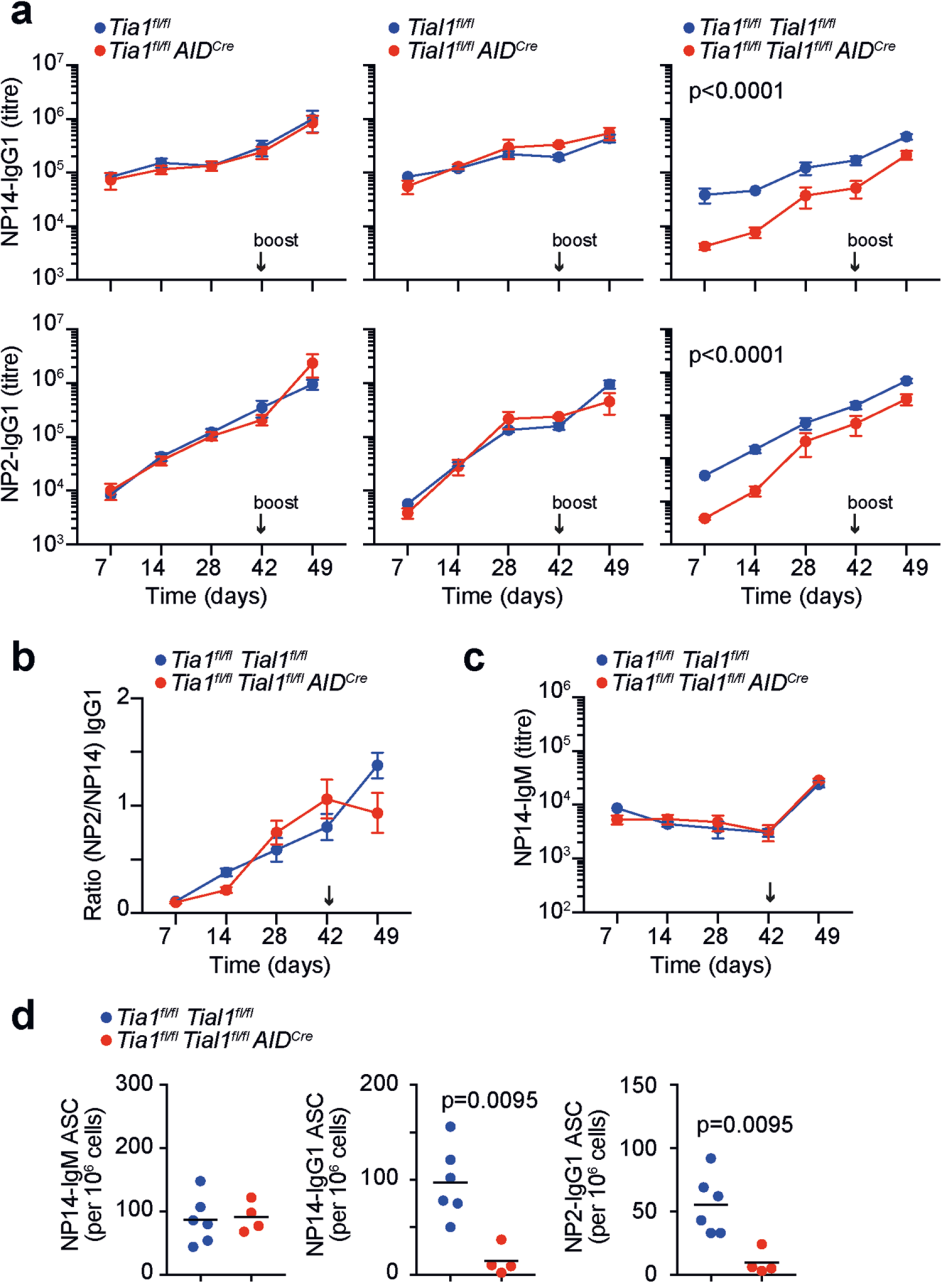
TIA1 and TIAL1 are required for class-switch and high-affinity antibody production. **a** Total NP14-IgG1 antibody titers in the serum of control, *Tia1*^*fl/fl*^ *AID*^*Cre*^*, Tial1*^*fl/fl*^ *AID*^*Cre*^ and *Tia1*^*fl/fl*^ *Tial1*^*fl/fl*^ *AID*^*Cre*^ mice immunized with NP-KLH in alum. **b** Ratio of high-affinity NP2-IgG1 and total NP14-IgG1 antibodies in the serum of control and *Tia1*^*fl/fl*^ *Tial1*^*fl/fl*^ *AID*^*Cre*^ mice from (**a**). **c** Serum titers of total NP14-IgM in mice from (**a**). Data shown in (**a**), (**b**) and (**c**) are from one of the two independent experiments performed with n = 5-8 mice per group. Data are shown as the mean ± SEM, 2-way ANOVA and Bonferroni’s post-test (**p < 0.01, ***p < 0.001). **d** Number of NP14-IgM-, NP14-IgG1- and high-affinity NP2-IgG1-specific antibody-secreting cells (ASCs) in the spleens of *Tia1*^*fl/fl*^ *Tial1*^*fl/fl*^ *AID*^*Cre*^ mice at Day 14 after immunization. Data are representative of one of the two independent experiments performed with n = 4-6 mice per group. Mann‒Whitney tests

Immunization of single or double KO mice for TIA1 and TIAL1 with the thymus-dependent antigen NP-KLH in alum followed by analysis of total (NP14-) and high-affinity (NP2-) antibodies in the serum of these mice revealed that both TIA1 and TIAL1 were required for the production of high-affinity class-switched antibodies (Fig. 2a). The *Tia1*^*fl/fl*^ *Tial1*^*fl/fl*^ *AID*^*Cre*^ mice had a 5- to 10-fold reduction in NP14-binding IgG1 serum titers compared to the littermate controls. In contrast, no differences were found in the *Tia1*^*fl/fl*^ *AID*^*Cre*^ and *Tial1*^*fl/fl*^ *AID*^*Cre*^ mice after both primary and secondary immunization, suggesting that TIA1 and TIAL1 have redundant functions (Fig. 2a). Flow cytometry analysis of TIA1 and TIAL1 expression in single KO GC B cells revealed that deletion of TIA1 was compensated by an increase in TIAL1, and vice versa (Supplementary Fig. 1c). This finding highlights the existence of a mutual cross-regulatory mechanism that controls the overall expression of TIA1 and TIAL1, as previously suggested [37].

Serum analysis of high-affinity NP2-binding IgG1 antibodies, which increase over time in C57BL/6 mice as a result of affinity maturation, showed no differences between the control, *Tia1*^*fl/fl*^ *AID*^*Cre*^ and *Tial1*^*fl/fl*^ *AID*^*Cre*^ mice, but they were highly reduced in the *Tia1*^*fl/fl*^ *Tial1*^*fl/fl*^ *AID*^*Cre*^ mice (Fig. 2a). Quantitation of the ratio between NP2- and NP14-binding IgG1 antibodies revealed no significant differences between the different mouse genotypes (Fig. 2b). This finding suggests that deletion of TIA1 and TIAL1 likely has a general impact on GC-dependent production of antibodies rather than on the selection of B-cell clones producing high-affinity antibodies.

The amount of serum NP14-binding IgM antibodies and the number of splenic antibody-secreting cells (ASCs) producing NP-specific IgM antibodies were not altered in the *Tia1*^*fl/fl*^ *AID*^*Cre*^, *Tial1*^*fl/fl*^ *AID*^*Cre*^ mice or *Tia1*^*fl/fl*^ *Tial1*^*fl/fl*^ *AID*^*Cre*^ mice compared to the control mice (Fig. 2c and d and Supplementary Fig. 2a and b). In contrast, the amount of ASC producing NP-specific IgG1 antibodies was diminished approximately 10-fold in the *Tia1*^*fl/fl*^ *Tial1*^*fl/fl*^ *AID*^*Cre*^ mice (Fig. 2d). Altogether, our data show that the expression of TIA1 and TIAL1 in GC B cells is required for the generation of ASCs producing high-affinity class-switched antibodies.

### TIA1 and TIAL1 are essential for the GC reaction

**Fig. 3:**
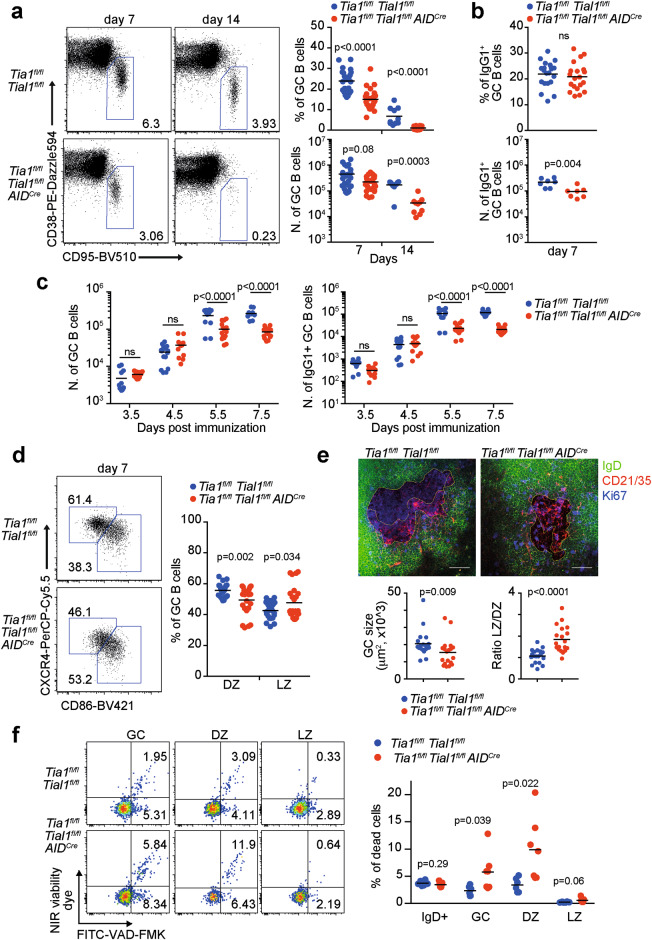
GC responses are impaired in the absence of TIA1 and TIAL1. **a** Analysis by flow cytometry of GCs in the spleen of control and *Tia1*^*fl/fl*^ *Tial1*^*fl/fl*^ *AID*^*Cre*^ mice 7 and 14 days post-immunization with NP-KLH in alum. Left panels, representative dot plots. Right panels, quantitation of the percentage and number of GC B cells. **b** Percentage and number of IgG1+ GC B cells in the spleen of control and *Tia1*^*fl/fl*^ *Tial1*^*fl/fl*^ *AID*^*Cre*^ mice 7 days after immunization. **c** Time course analysis of total and IgG1+ GC B cells in draining LNs from control and *Tia1*^*fl/fl*^ *Tial1*^*fl/fl*^ *AID*^*Cre*^ mice. **d** Distribution of GC B cells in the DZ and LZ analyzed by flow cytometry (CXCR4^+^ CD86^-^ DZ GC B cells and CXCR4^-^ CD86^+^ LZ GC B cells). **e** Visualization of GCs in the spleen of control and *Tia1*^*fl/fl*^ *Tial1*^*fl/fl*^ *AID*^*Cre*^ mice by confocal microscopy. Bottom panels show the size and LZ/DZ ratio in each of the 19 GCs analyzed from 4 different mice analyzed in two independent experiments. **f** Representative pseudocolor dot plots showing GC cell viability by flow cytometry. Proapoptotic and apoptotic cells were labeled with FITC-VAD-FMK in combination with the Zombie NIR fixable viability dye. Data in (**a**), (**b**), (**c**) and (**d**) are from two to four independent experiments performed with at least *n* = 5 mice per group. Data in (**f**) are representative of one of the three independent experiments performed. Mann‒Whitney tests were performed for statistical analysis

Time-course quantification of GC responses in the spleen of the mice immunized with NP-KLH revealed a progressive loss of GC B cells in the *Tia1*^*fl/fl*^ *Tial1*^*fl/fl*^ *AID*^*Cre*^ mice compared to the control mice (Fig. 3a). At Day 7, early GC responses were reduced 2-fold in the absence of TIA1 and TIAL1. At Day 14, the percentage and number of GC B cells were decreased in the *Tia1*^*fl/fl*^ *Tial1*^*fl/fl*^ *AID*^*Cre*^ mice by more than 10-fold. Interestingly, single conditional deletion of *Tia1* or *Tial1* in GC B cells had no impact on the formation and maintenance of GCs (Supplementary Fig. 2c, d). This finding confirms that TIA1 and TIAL1 are both essential for the development of GCs in response to foreign antigens.

Further characterization of defective GCs in the *Tia1*^*fl/fl*^ *Tial1*^*fl/fl*^ *AID*^*Cre*^ mice showed no differences in the percentage of IgG1^+^ GC B cells compared to that of the control mice at Day 7 after immunization (Fig. 3b). In contrast, the number of IgG1^+^ GC B cells was reduced 2-fold, in line with the overall reduction of the GC compartment in the *Tia1*^*fl/fl*^ *Tial1*^*fl/fl*^ *AID*^*Cre*^ mice, indicating that TIA1 and TIAL1 could be dispensable for Ig class-switch recombination (CSR) in B cells. CSR mostly occurs outside GCs early upon immunization [38]. Therefore, we measured the appearance and expansion of IgG1+ GC B cells in the LNs of the control and *Tia1*^*fl/fl*^ *Tial1*^*fl/fl*^ *AID*^*Cre*^ mice immunized with NP-KLH (Fig. 3c). Early GC seeding and generation of IgG1^+^ GC B cells were unaffected in the *Tia1*^*fl/fl*^ *Tial1*^*fl/fl*^ *AID*^*Cre*^ mice. TIA1 and TIAL1 were efficiently deleted in GC B cells at Days 3.5 and 4.5 after immunization, reinforcing the notion that these proteins are not needed for Ig CSR (Supplementary Fig. 1b). The number of both total and IgG1^+^ GC B cells was significantly decreased after 5.5 days of immunization (Fig. 3c), confirming our previous results from spleens. Next, we tested whether deletion of TIA1 and TIAL1 in the *AID*^*Cre*^ model altered the capacity of B cells to undergo CSR in an in vitro-derived GC B-cell culture system. First, we confirmed that these RBPs were efficiently depleted in IgG1^+^ *Tia1*^*fl/fl*^ *Tial1*^*fl/fl*^ *AID*^*Cre*^ iGC B cells (Supplementary Fig. 3a). Then, quantitation of iGC B-cell expansion and IgG1^+^ iGC B cells in these cultures revealed no differences based on the cell genotype (Supplementary Fig. 3b). Taken together, our data suggest that TIA1 and TIAL1 are dispensable for Ig class-switch recombination (CSR) in B cells.

Assessment of the GC B-cell phenotype based on the cell surface expression of CD86 and CXCR4 revealed a significant reduction in the percentage of dark zone (DZ) GC B cells and a concomitant increase in the percentage of light zone (LZ) GC B cells in the *Tia1*^*fl/fl*^ *Tial1*^*fl/fl*^ *AID*^*Cre*^ mice compared to the control mice (Fig. 3d). Confocal imaging confirmed the reduction in the size of GCs found in the spleen of the *Tia1*^*fl/fl*^ *Tial1*^*fl/fl*^ *AID*^*Cre*^ mice as well as the altered distribution of GC cells between the DZ and the LZ (Fig. 3e). DZ GC B cells undergo several rounds of proliferation and Ig SHM that expand the diversity of antibodies but also cause metabolic and replicative stress leading to cell death. Indeed, quantitation of apoptotic GC B cells using the caspase inhibitor VAD-FMK labeled with FITC showed an increased percentage of nonviable DZ GC B cells when compared to LZ GC B cells in the control mice (Fig. 3f). Importantly, the percentage of nonviable GC B cells was increased by 2-fold in the *Tia1*^*fl/fl*^ *Tial1*^*fl/fl*^ *AID*^*Cre*^ mice compared to the control mice. This result was mostly due to the important loss of cell viability of DZ GC B cells (Fig. 3f). Thus, we conclude that TIA1 and TIAL1 are essential for the survival and maintenance of GC B cells.

### TIA1 and TIAL1 are required for the survival of antigen-specific GC B cells

**Fig. 4:**
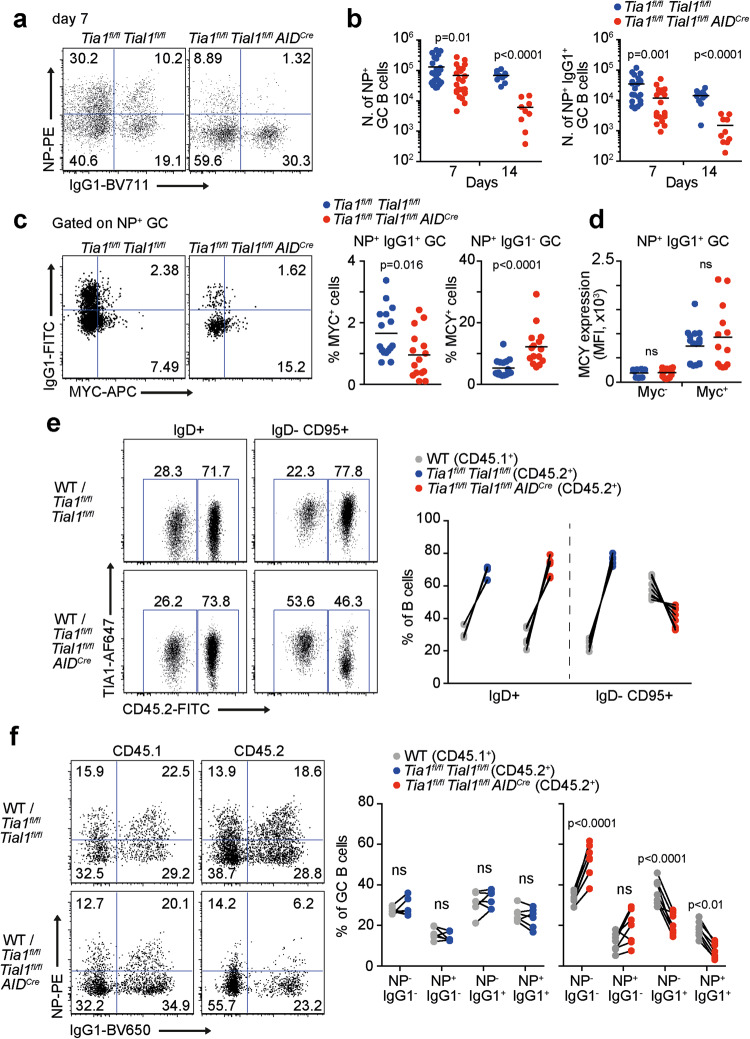
TIA1 and TIAL1 are essential for the expansion of antigen-specific GC B cells. **a** Representative flow cytometry dot plots for quantification of IgG1 class-switched NP-specific GC B cells. **b**, Number of NP antigen-specific GC B cells in the spleen of control and *Tia1*^*fl/fl*^ *Tial1*^*fl/fl*^ *AID*^*Cre*^ mice after 7 and 14 days of immunization with NP-KLH. Data are from three independent experiments performed with at least 5 mice per genotype. **c** Analysis of positively selected MYC^+^ NP^+^ GC B cells in control and *Tia1*^*fl/fl*^ *Tial1*^*fl/fl*^ *AID*^*Cre*^ mice. **d** Median fluorescence intensity (MFI) of MYC in positively selected NP^+^ IgG1^+^ GC B cells. Data in c and d are from two independent experiments performed each with at least 6 mice per genotype. **e** Analysis of IgD^+^ and GC B cells in the spleen of bone marrow (BM) chimeric mice at Day 10 after immunization with NP-KLH. Left panels, representative dot plots showing expression of the congenic marker CD45.2 and TIA1 expression. Right panel, percentage of IgD^+^ and GC B cells in BM chimeric mice originating from the BM of wild-type (CD45.1), control (CD45.2) or *Tia1*^*fl/fl*^ *Tial1*^*fl/fl*^ *AID*^*Cre*^ (CD45.2) mice. **f** Quantitation of antigen-specific GC B cells in the spleen of BM chimeric mice. Congenic markers (CD45.1 and CD45.2) were used to assess cell origin. Left panels, representative dot plots. Right panels, percentage of class-switched GC B cells recognizing NP antigen. Data from (**e**) and (**f**) are representative of one of the two independent experiments performed with n = 6 mice per group. Mann‒Whitney tests were performed in (**b**), (**c**) and (**d**). Paired t tests were performed in (**f**)

Generation of antigen-specific GC B cells was severely impaired in the absence of TIA1 and TIAL1 (Fig. 4a and b, and Supplementary Fig. 3c). Total NP^+^ GC B cells were decreased in the *Tia1*^*fl/fl*^ *Tial1*^*fl/fl*^ *AID*^*Cre*^ mice at Day 7 post-immunization. The number of these antigen-specific GC B cells declined further as the GC reaction progressed based on positive selection. IgG1- switched NP^+^ GC B cells were particularly impacted and reduced by more than 10-fold in the *Tia1*^*fl/fl*^ *Tial1*^*fl/fl*^ *AID*^*Cre*^ mice (Fig. 4a and b). The generation of NP^+^ IgG1^+^ memory B cells was also reduced in the absence of TIA1 and TIAL1 (Supplementary Fig. 3d, e), consistent with the defective memory antibody responses found in the *Tia1*^*fl/fl*^ *Tial1*^*fl/fl*^ *AID*^*Cre*^ mice (Fig. 2a).

MYC is upregulated in GC B cells upon positive selection to drive further rounds of GC B-cell proliferation and SHM [10, 11]. Analysis of the percentage of MYC^+^ GC B cells revealed a preferential selection of nonclass-switched GC B cells in the *Tia1*^*fl/fl*^ *Tial1*^*fl/fl*^ *AID*^*Cre*^ mice, whereas the percentage of NP^+^ IgG1^+^ GC B cells expressing MYC was reduced by almost 2-fold (Fig. 4c). MYC protein expression was unchanged when comparing MYC^+^ NP^+^ IgG1^+^ GC B cells from the control and *Tia1*^*fl/fl*^ *Tial1*^*fl/fl*^ *AID*^*Cre*^ mice (Fig. 4d). Therefore, we concluded that TIA1 and TIAL1 are required for positive selection of GC B cells, likely in a MYC-independent manner.

To interrogate whether TIA1 and TIAL1 expression was required for antigen-specific GC B-cell fitness, we reconstituted the lymphoid compartment of Rag2 KO mice by transferring bone marrow (BM) from the wild-type (CD45.1^+^) and *Tia1*^*fl/fl*^ *Tial1*^*fl/fl*^ (CD45.2^+^) or *Tia1*^*fl/fl*^ *Tial1*^*fl/fl*^ *AID*^*Cre*^ (CD45.2^+^) mice at a ratio of 1:4 (Fig. 4e and Supplementary Fig. 3f). Analysis of GC responses in these chimeric mice at Day 10 after immunization showed a reduction in the percentage of *Tia1 Tial1* dKO CD45.2^+^ GC B cells and a concomitant expansion of control CD45.1^+^ GC B cells (Fig. 4e). NP-specific and class-switched GC B cells lacking the expression of TIA1 and TIAL1 were specifically outcompeted in these chimeric mice (Fig. 4f). *Tia1 Tial1* dKO NP^+^ IgG1^+^ CD45.2^+^ GC B cells represented only 5% of the total number of GC B cells, in deep contrast to the 20% achieved by control NP^+^ CD45.1^+^ GC B cells. Similarly, the percentage of NP^-^ IgG1^+^ *Tia1 Tial1* dKO GC B cells was reduced by 2-fold compared to that of control cells. Thus, TIA1 and TIAL1 deletion confers a competitive disadvantage for the selection, expansion and survival of antigen-specific GC B cells.

### TIA1 and TIAL1 enforce the identity of GC B cells

**Fig. 5:**
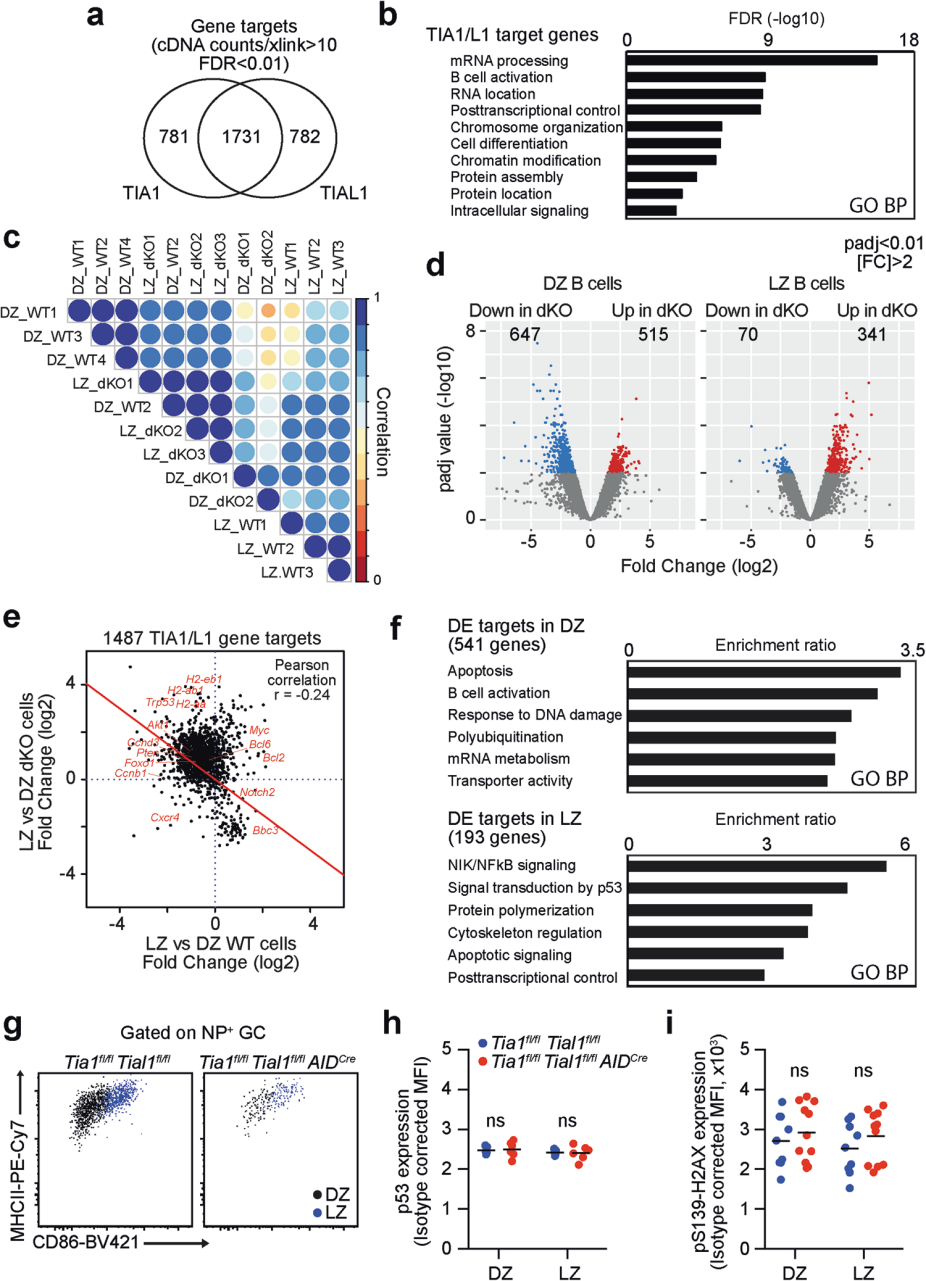
TIA1 and TIAL1 confer transcriptional identity to DZ and LZ B cells. **a** Venn diagram showing the number of TIA1 and TIAL1 target genes identified with high confidence in iCLIP assays (FDR < 0.01, at least 10+ cDNA counts annotated to 1 or more crosslink sites). **b** Gene Ontology enrichment analysis showing the top 10 pathways to which TIA1 and TIAL1 gene targets are associated. **c** Correlation plot showing the Euclidian distance between the transcriptomes of DZ and LZ B cells sorted from control and *Tia1*^*fl/fl*^ *Tial1*^*fl/fl*^ *AID*^*Cre*^ mice. **d** Volcano plots showing the number of differentially expressed genes in DZ and LZ B cells and *Tia1 Tial1* dKO DZ and LZ B cells when compared to their control counterparts (DESeq2 analyses, padj<0.01, [fold change]>2). **e** Analysis of the expression changes of TIA1 and TIAL1 target genes in LZ versus DZ B cells in control and *Tia1*^*fl/fl*^ *Tial1*^*fl/fl*^ *AID*^*Cre*^ mice. **f** Top pathways enriched with differentially expressed TIA1 and TIAL1 target genes in *Tia1 Tial1* dKO DZ and LZ cells. **g** Representative dot plot showing the expression of MHC class II in DZ and LZ B cells from control and *Tia1*^*fl/fl*^ *Tial1*^*fl/fl*^ *AID*^*Cre*^ mice. **h** Quantitation by flow cytometry of p53 expression in GC B cells from control and *Tia1*^*fl/fl*^ *Tial1*^*fl/fl*^ *AID*^*Cre*^ mice at Day 7 after immunization with NP-KLH. Data are shown as the corrected MFI from 1 of the 2 independent experiments performed with n = 5 and 6 per genotype. **i** Analysis by flow cytometry of pSer139-H2A.X expression in GC B cells from control and *Tia1*^*fl/fl*^ *Tial1*^*fl/fl*^ *AID*^*Cre*^ mice at Day 7 after immunization. Data are from two independent experiments performed with at least 5 mice per group. Mann‒Whitney statistical tests in h and i

To uncover the molecular mechanisms controlled by TIA1 and TIAL1 in GC B cells, we combined global transcriptomics analyses with the identification of TIA1 and TIAL1 RNA targets using UV-crosslinking and immunoprecipitation (iCLIP) in B cells stimulated with αIgM, αCD40 and IL4 that mimicked signaling received during GC positive selection. Comparison of TIA1 and TIAL1 iCLIP assays revealed a high overlap between the sites of interaction and the genes targeted by these RBPs in B cells (Fig. 5a, Supplementary Fig. 4 and Supplementary Table 1). This finding is in line with a previous report in HeLa cells [39] and highlights the functional redundancy between TIA1 and TIAL1. The density and overlap binding of TIA1 and TIAL1 was greatest in 3'UTRs, although these RBPs were also found to be extensively bound to introns (Supplementary Fig. 4b, c, d). RNA transcripts from 1731 genes were targeted by both TIA1 and TIAL1 with a high degree of confidence (Fig. 5a and Supplementary Fig. 4e), with another 781 and 782 genes being targeted by TIA1 and TIAL1, respectively. Gene Ontology analyses showed that these genes were mostly involved in B-cell activation and processing of RNAs (Fig. 5b), highlighting that TIA1 and TIAL1 might indeed control the expression of specific RNA targets during GC B-cell positive selection.

Analysis of the transcriptome of DZ and LZ GC B cells sorted from the control and *Tia1*^*fl/fl*^ *Tial1*^*fl/fl*^ *AID*^*Cre*^ mice revealed the profound impact of TIA1 and TIAL1 deletion in GCs (Fig. 5c and Supplementary Table 2). As expected, control DZ and LZ GC B cells had a highly distinctive transcriptome, characteristic of their diverse functions. However, the transcriptome of *Tia1 Tial1* dKO DZ B cells correlated more closely with that of LZ GC B cells, and this was reflected in the total number of differentially expressed (DE) genes in the *Tia1 Tial1* dKO DZ and LZ B cells compared to their counterpart control cells (1162 DE genes in *Tia1 Tial1* dKO DZ cells vs. 411 DE genes in *Tia1 Tial1* dKO LZ cells) (Fig. 5d). Further comparisons of how genes were differentially expressed in DZ and LZ cells from the control and *Tia1*^*fl/fl*^ *Tial1*^*fl/fl*^ *AID*^*Cre*^ mice consistently showed a negative correlation, suggesting that *Tia1 Tial1* dKO GC B cells failed to modulate the expression of genes during the LZ to DZ transition (Supplementary Fig. 5a). Indeed, we found an inverse correlation between the number of DE genes in *Tia1 Tial1* dKO LZ and DZ B cells compared to their counterpart control cells (Fig. 5d). The RNA abundance of 341 genes was increased in *Tia1 Tial1* dKO LZ cells, while only 70 genes were downregulated. In contrast, 647 genes were decreased in *Tia1 Tial1* dKO DZ cells, while 515 genes were increased (Fig. 5d). The vast majority of the 1487 TIA1 and TIAL1 target genes identified with high confidence using iCLIP and expressed by GC B cells failed to be regulated in *Tia1 Tial1* dKO DZ cells after transition from the LZ (Fig. 5e). While genes of the MHC class II complex that support antigen recognition remained highly expressed in *Tia1 Tial1* dKO DZ cells, genes encoding signaling molecules and cell cycle mediators required for signal transduction shutdown and cycling of GC B cells after positive selection failed to be upregulated in *Tia1 Tial1* dKO DZ cells (e.g., *Pten*, *Foxo1, Ccnb1 and Ccnd3)* (Fig. 5e). Indeed, DE genes targeted by TIA1 and TIAL1 were mostly associated with these pathways as well as with those linked to ROS metabolism, responses to DNA damage, RNA metabolism and apoptosis (Fig. 5f, Supplementary Fig. 5b and Supplementary Table 2). In summary, together with flow cytometry and imaging data, our transcriptomics analyses highlight the need for TIA1 and TIAL1 for GC B-cell distribution in the LZ and DZ and the transcriptome remodeling that confers cell identity to LZ and DZ GC B cells after positive selection.

### TIA1 and TIAL1 are dispensable for DNA damage control and proliferation of GC B cells

Flow cytometry analyses revealed that the expression of MHC class II was unaltered in *Tia1 Tial1* dKO GC B cells (Fig. 5g). Similarly, we did not find any defect in the expression and activation of key components of the PI3K pathway (Supplementary Fig. 5c), suggesting that the capacity to capture and present antigens and activation remains normal in GC B cells lacking TIA1 and TIAL1.

Pathway analyses indicated that ROS metabolism, DNA damage and signal transduction by p53 were altered in *Tia1 Tial1* dKO GC B cells. Indeed, we and others have previously shown that TIA1 and TIAL1 might control mitochondria and energy metabolism [40, 41], as well as p53 mRNA translation [42] and DNA damage responses [22, 26]. Thus, we assessed global protein synthesis in vivo as a measure of energy metabolism, phosphorylation of histone H2A.X as a marker of DNA damage, and p53 expression. Puromycin labeling of nascent peptides showed an increase in protein synthesis in metabolically active GC B cells in comparison to naïve IgD^+^ B cells and memory B cells (Supplementary Fig. 6a). However, we observed no differences between control and *Tia1 Tial1* dKO GC B cells in their capacity to synthesize proteins or in translation elongation measured after blocking the incorporation of new ribosomes into the start codon with harringtonine (Supplementary Fig. 6b). Quantitation of p53 and pSer139-H2A.X in GC B cells from the control and *Tia1*^*fl/fl*^ *Tial1*^*fl/fl*^ *AID*^*Cre*^ mice again showed no differences in the expression of these two markers of DNA damage and apoptosis (Fig. 5h, i). Taken together, these data indicate that TIA1 and TIAL1 are largely dispensable for total protein synthesis, energy metabolism, p53 expression or DNA damage detection and repair in GC B cells.

TIA1 and TIAL1 have been associated with the timely expression of MYC and in vitro clonal expansion in several cell lines [20, 21, 43, 44]. Despite the fact that *Myc* mRNA abundance was not altered in *Tia1 Tial1* dKO GC B cells (Supplementary Table 2), we investigated whether MYC activity as a transcription factor (TF) was altered in these cells. By combining our transcriptomics data and TF networks, we could infer TF activities in GC B cells based on changes in gene expression [35]. MYC activity was found to be increased in LZ GC B cells from *Tia1*^*fl/fl*^ *Tial1*^*fl/fl*^ *AID*^*Cre*^ mice compared to control cells (Supplementary Fig. 6c). This finding reinforced the notion that the defects found in *Tia1 Tial1* dKO GC B cells were likely independent of MYC.

Next, we assessed whether TIA1 and TIAL1 were required for GC B-cell cycling. Short-term labeling of DNA in cycling cells with BrdU showed no differences in proliferation between GC B cells from control and *Tia1*^*fl/fl*^ *Tial1*^*fl/fl*^ *AID*^*Cre*^ mice (Supplementary Fig. 6d). Further analysis of the percentage of GC B cells present at each stage of the cell cycle again revealed no differences. Therefore, we conclude that TIA1 and TIAL1 are not needed for the cell cycle in GC B cells.

### TIA1 and TIAL1 are required for MCL1 expression and GC B-cell survival

**Fig. 6:**
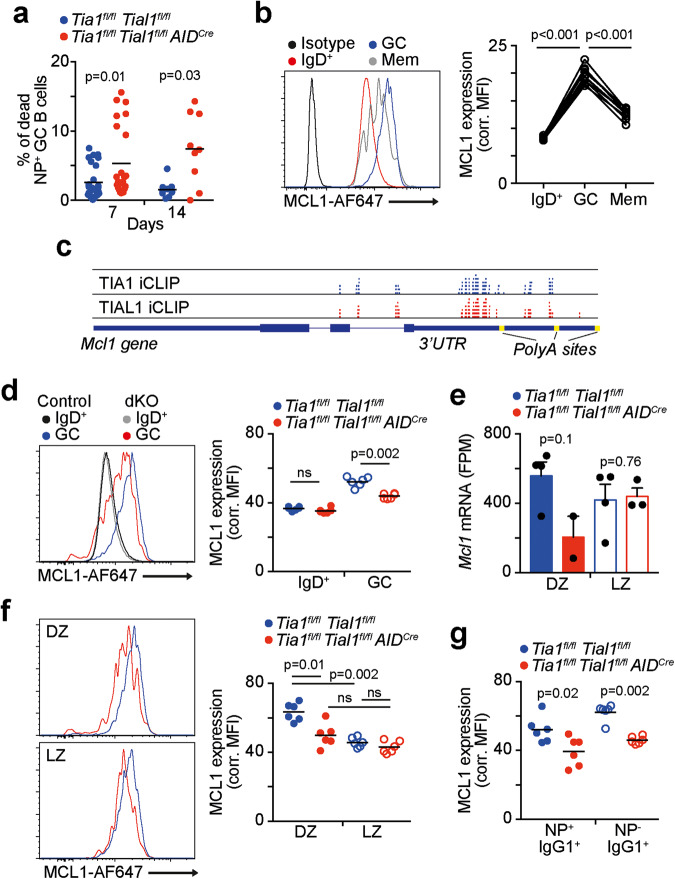
TIA1 and TIAL1 control the expression of MCL1 and survival of GC B cells. **a** Viability of antigen-specific GC cells in control and *Tia1*^*fl/fl*^ *Tial1*^*fl/fl*^ *AID*^*Cre*^ mice at Days 7 and 14 post-immunization with NP-KLH. Cell viability was assessed using FITC-VAD-FMK and Zombie NIR viability dye. Data are from two or three independent experiments depending on the time point with at least five mice used per genotype and experiment. **b** Analysis by FACS of MCL1 expression in naïve, GC and memory B cells. Data from one of the four independent experiments performed are shown (n = 7 mice). **c** Genome viewer of TIA1 and TIAL1 binding sites annotated to *Mcl1* mRNA. Polyadenylation sites with a central AAUAAA sequence are marked in yellow. **d** Quantitation of MCL1 expression in naïve and GC B cells from control and *Tia1*^*fl/fl*^ *Tial1*^*fl/fl*^ *AID*^*Cre*^ mice. Data from one of the four independent experiments performed with at least 5 mice per genotype. **e** *Mcl1* mRNA abundance in DZ and LZ B cells from control and *Tia1*^*fl/fl*^ *Tial1*^*fl/fl*^ *AID*^*Cre*^ mice. Data from the mRNAseq analyses are shown in Fig. 5c. P-adjusted values are from differential expression analyses with DESeq2. **f** MCL1 expression in DZ and LZ B cells from the mice shown in (**d**). **g** Expression of MCL1 in IgG1-class switched GC B cells in mice from (**d**). Mann‒Whitney tests were performed in (**a**), (**d**), (**f**) and (**g**). Paired t tests were performed for statistical analysis in (**b**)

Apoptosis was the common pathway found to be deregulated in both *Tia1 Tial1* dKO DZ and LZ cells. Indeed, we found a higher proportion of NP^+^ GC B cells dying in *Tia1*^*fl/fl*^ *Tial1*^*fl/fl*^ *AID*^*Cre*^ mice than in control mice at both Days 7 and 14 upon immunization (Fig. 6a). GC B cells rely solely on the expression of the BCL2 family member MCL1 for cell survival after positive selection [45]. MCL1 expression was increased in NP^+^ GC B cells compared to naïve or NP^+^ memory B cells (Fig. 6b). Importantly, iCLIP assays showed that TIA1 and TIAL1 were extensively associated with *Mcl1* transcripts (Fig. 6c) and enabled MCL1 protein synthesis in GC B cells (Fig. 6d). We found no differences in the mRNA abundance of *Mcl1* in LZ B cells from the control and *Tia1*^*fl/fl*^ *Tial1*^*fl/fl*^ *AID*^*Cre*^ mice; however, there was a tendency toward a reduction in *Tia1 Tial1* dKO DZ B cells (Fig. 6e). Interestingly, protein quantification by flow cytometry showed that DZ B cells had higher levels of MCL1 protein than LZ B cells. In contrast, *Tia1 Tial1* dKO DZ B cells failed to significantly increase the levels with respect to LZ B cells (Fig. 6f). Similarly, *Tia1 Tial1* dKO IgG1-class switched GC B cells showed a significant reduction in the expression of MCL1 when compared to control cells (Fig. 6g). Altogether, our data indicate that TIA1 and TIAL1 are key post-transcriptional promoters of MCL1 protein synthesis and survival in GC B cells.

### TIA1 and TIAL1 control *Mcl1* mRNA translation

**Fig. 7:**
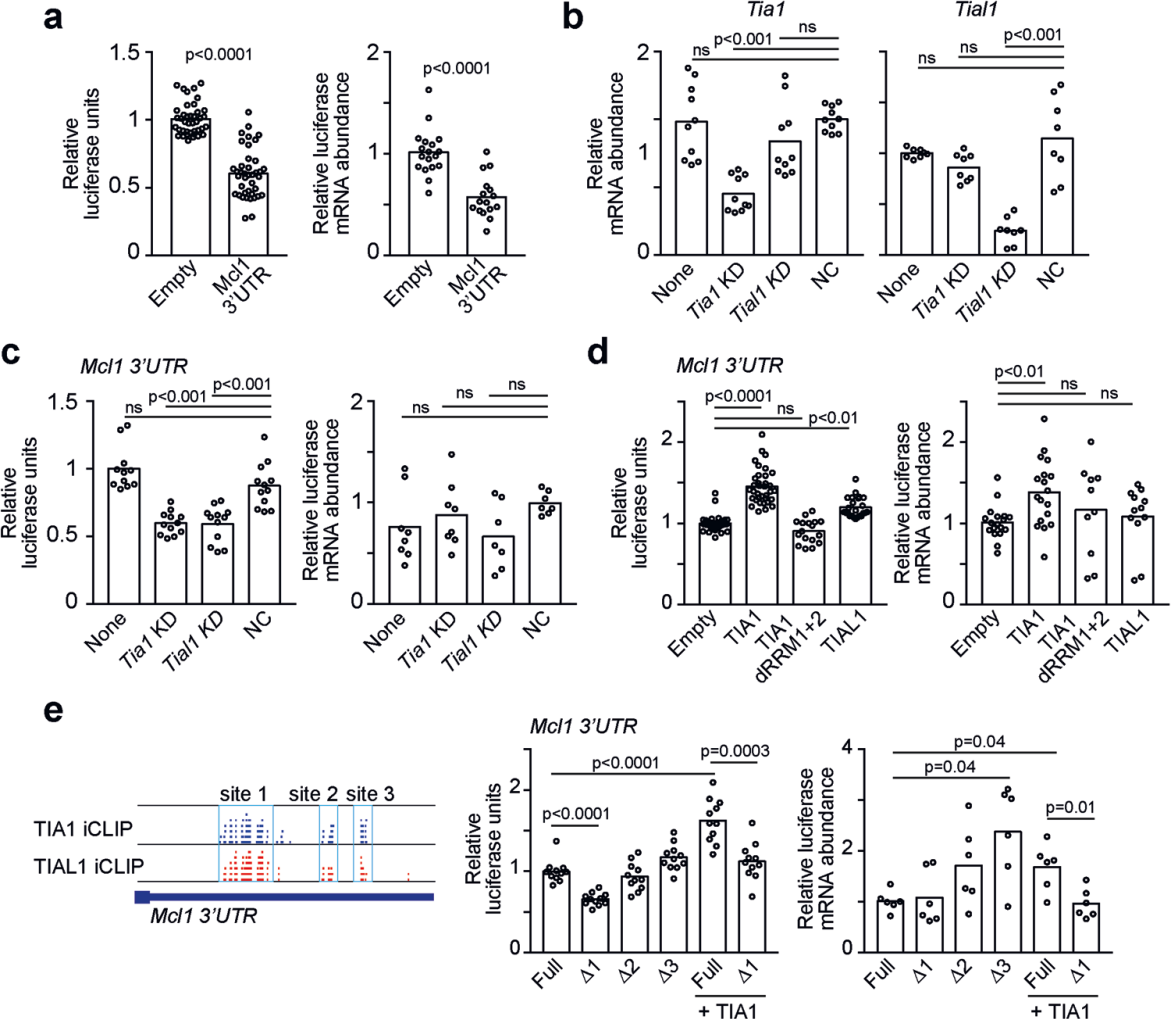
TIA1 and TIAL1 control *Mcl1* mRNA translation. **a** Quantitation of Renilla luciferase mRNA abundance (right) and protein signal (left) in HEK293T cells transfected with a control dual luciferase reporter psiCheck2-empty or a psiCheck2- *Mcl1* 3'UTR. **b** Analysis of *Tia1* and *Tial1* mRNA levels in cells transfected with specific DsiRNAs. **c** Renilla luciferase mRNA and protein abundance in HEK293T cells upon transient depletion of TIA1 or TIAL1. **d** Measurement of Renilla luciferase upon overexpression of TIA1, TIAL1 or a mutant TIA1 lacking RNA recognition motifs 1 and 2 (TIA1 dRRM1 + 2). **e** Identification of the TIA:RNA binding sites deleted in the dual luciferase reporter psiCheck2- *Mcl1* 3'UTR. Mid and right panels, quantitation of luciferase RNA and protein abundance. Data from at least three independent experiments performed with a minimum of two replicates are shown in all figure panels. Relative luciferase units were calculated by normalizing the Renilla luciferase signal to the control firefly luciferase signal and were relatively quantified to the control group. Mann‒Whitney tests were performed for statistical analysis

To further understand the molecular mechanism by which TIA1 promotes MCL1 expression, we used a dual luciferase reporter system in which the *Mcl1* 3'UTR was placed downstream of the stop codon of the Renilla luciferase coding sequence. The *Mcl1* 3'UTR conferred transcript instability to the luciferase reporter, reducing the synthesis of protein (Fig. 7a). Knockdown of TIA1 and TIAL1 expression (Fig. 7b) did not affect the mRNA abundance of this luciferase reporter; however, it significantly decreased protein synthesis (Fig. 7c). Conversely, overexpression of TIA1, and to a lesser extent TIAL1, led to a highly significant increase in reporter protein expression (Fig. 7d). This change was accompanied by a mild increase in mRNA abundance only when TIA1 was overexpressed. Importantly, overexpression of a mutant form of TIA1 lacking the RNA recognition motifs that was therefore incapable of binding to RNA had no effect on the expression of the luciferase reporter (Fig. 7d). To confirm that TIA1 binding to the *Mcl1* 3'UTR indeed controlled mRNA translation, we deleted the three most prominent RNA binding sites of TIA1 and TIAL1 in the *Mcl1* 3'UTR (Fig. 7e). Quantitation of the luciferase RNA and protein showed a significant reduction in protein synthesis upon deletion of TIA-binding site 1. This effect was independent of any changes in RNA abundance, suggesting that the protein:RNA interactions at this site are responsible for the regulation of mRNA translation (Fig. 7e). Deletion of TIA-binding site 1 also reversed the increase in the luciferase reporter promoted by TIA1 overexpression. In contrast, deletion of TIA-binding sites 2 and 3 did not lead to any changes in luciferase protein expression but mildly increased the overall mRNA abundance, indicating that they are responsible for *Mcl1* mRNA destabilization in a TIA-independent manner (Fig. 7e). Taken together, our data demonstrate that direct binding of TIA1 and TIAL1 to the *Mcl1* 3'UTR promotes the translation and synthesis of MCL1.

## Discussion

Emerging evidence highlights the essential roles of RBPs during pathogenic responses. In this study, we show that the RBPs TIA1 and TIAL1 shape the transcriptome of GC B cells to provide cell identity and allow cell selection upon antigen recognition. GC and antibody responses are impaired in the absence of TIA1 and TIAL1. They are dispensable for B-cell CSR but essential for the positive selection and survival of antigen-specific GC B cells.

In this study, we found that TIA1 protein expression is increased in GC B cells compared to FO B cells. The expression of TIAL1 remained unchanged; however, deletion of both proteins was required to impair GC responses. Previous reports have shown that TIA1 and TIAL1 control the expression of each other in cell lines [37], and this compensatory mechanism was also observed in GC B cells from single cKO mice. More important than changes in protein abundance is the fact that the binding capacity of TIA1 can be modulated in response to intracellular and external cues [22, 26]. Here, we show that TIA1 and TIAL1 bind to the same RNA binding sites and act redundantly over their RNA targets, as previously shown in different cancer cell lines [39, 43]. Protein‒RNA interaction analyses with iCLIP could not be performed in ex vivo purified GC B cells due to cell number limitations, but it is tempting to hypothesize that TIA-RNA binding capacity could differ between GC B cells based on their AID activity or upon encountering Tfh cells.

Here, we provide convincing evidence that TIA1 and TIAL1 modulate MCL1 expression at the post-transcriptional level. MCL1 is the only BCL2 family member required for the survival of GC B cells [45]. MCL1 is needed throughout B-cell development [46] and is often deregulated in B-cell lymphomas with a GC origin [47, 48]. *Mcl1* mRNA abundance does not change between different GC B-cell subsets or upon antigen encounter and selection [49, 50]. However, we found that MCL1 protein is significantly increased in DZ B cells in a transcription-independent manner. The DZ is the microanatomic site for selection against deleterious mutations introduced by AID [51], in which expression of MCL1 will be required to preserve cells from dying. In *Tia1 Tial1 AID*^*Cre*^ mice, DZ B cells fail to upregulate the expression of MCL1, indicating that TIA1 and TIAL1 can control the expression of this essential prosurvival factor in these cells. Our iCLIP data showed that TIA1 and TIAL1 bind extensively within the *Mcl1* 3'UTR, and luciferase reporter assays demonstrated that TIA1 and TIAL1 promote *Mcl1* mRNA translation. In addition, it is possible that tight control of alternative mRNA polyadenylation is an important mechanism for MCL1 protein production [52]. Although we did not test whether this mechanism was altered in *Tia1 Tial1* dKO DZ GC B cells due to the rapid elimination of these cells, TIA1 and TIAL1 have been widely involved in the polyadenylation and timely translation of mRNA targets such as *eIF4E* that induce MCL1 expression in the cell [21, 53, 54]. Alternative usage of polyadenylation sites marks the production of membrane-bound or secreted immunoglobulins [55, 56], and the length of polyA tails differs between DZ and LZ B cells and those GC B cells terminally differentiating into ASCs [57]. Further studies should elucidate the importance of mRNA translation and polyadenylation in defining GC B-cell fate and identify RBPs, such as CstF-64 [58, 59], TIA1 and TIAL1, that might control this mechanism.

In addition to TIA-dependent regulation of MCL1, we revealed that deletion of TIA1 and TIAL1 has much broader consequences for the transcriptional identity of LZ and DZ B cells. Despite the fact that TIAL1 has been found to modulate *Myc* mRNA translation in different cell lines [20, 44] and that GC responses in *Tia1*^*fl/fl*^ *Tial1*^*fl/fl*^ *AID*^*Cre*^ mice are similar to those upon conditional deletion of *Myc*, with a strong reduction in the DZ compartment and with GC B cells unable to modulate the expression of featured genes of DZ and LZ B cells [10, 11], we found no evidence that MYC expression or MYC-associated gene signatures were altered in *Tia1 Tial1* dKO GC B cells. The number of antigen-specific MYC^+^ GC B cells was largely reduced in the *Tia1*^*fl/fl*^ *Tial1*^*fl/fl*^ *AID*^*Cre*^ mice. This phenomenon was independent of the capacity of GC B cells to express MHCII molecules for antigen presentation or to become activated upon Tfh cell encounter. This result suggested that genes downstream of the MYC activation program, such as MCL1, should be responsible for the loss of antigen-specific GC B cells in *Tia1*^*fl/fl*^ *Tial1*^*fl/fl*^ *AID*^*Cre*^ mice.

One possibility was that TIA1 and TIAL1 controlled cell cycle progression, as proposed previously in cell lines [43, 60]. We found that multiple cell cycle mediators were targets of TIA1 and TIAL1, with the expression of *Cdk2*, *Ccnb1*, *Ccnb2*, and other cell cycle-associated mRNAs being decreased in *Tia1 Tial1* dKO DZ B cells. Despite this, we did not detect any changes in GC B-cell progression through the cell cycle. This finding suggested that, as in progenitor B cells [25], TIA1 and TIAL1 do not regulate the cell cycle in GC B cells. TIA1- and TIAL1-dependent regulation of the cell cycle might then be specific to the conditions and cell types analyzed. Similarly, *Trp53* mRNA translation or DNA damage responses were unaltered in *Tia1 Tial1* dKO GC B cells. This result can be explained by the fact that in the *AID*^*Cre*^ mouse, conditional gene deletion only occurs after AID expression and induction of AID-mediated DNA damage. Taken together, our data reinforce the notion that cell identity, selection and survival, rather than DNA damage and proliferation, are the primary causes of GC loss in the *Tia1*^*fl/fl*^ *Tial1*^*fl/fl*^ *AID*^*Cre*^ mice.

TIA1 and TIAL1 likely cooperate with other RBPs to secure timely development of GC responses. We have previously shown that HuR and Ptbp1 are part of a post-transcriptional program for antigen-specific GC B-cell selection and expansion [8, 9]. TIA1, TIAL1 and HuR bind all to the same U-rich elements present in the 3'UTRs of mRNA targets such as *Myc* and *Mcl1*. Understanding the cooperation between HuR [61] and TIAL1 for *Mcl1* mRNA stabilization and translation, in particular, and how they compete with other RBPs (e.g., TTP [62] or PTBP1 [63]) and microRNAs [64] with antagonist functions will be highly relevant to explain the long-lasting persistence of GC and antibody responses. Differences in the kinetics of binding, affinity for binding sites and cooperation with other RBPs might define their importance for post-transcriptional regulation of each specific RNA target. A seeding study comparing these aspects in vitro showed that although HuR and TIAL1 bind to U-rich elements with nanomolar affinity, TIAL1 binding is more stable over time [65]. TIAL1 binding leads to an elongated protein:RNA shape in which HuR can also bind by surrounding the RNA molecule with its RNA recognition motifs [65–67]. Thus, it is possible that different RBPs bind to the same RNA targets and constitute a network for post-transcriptional gene regulation that allows GC development and maintenance.

## Supplementary information

Supplemental Figure 1

Supplemental Figure 2

Supplemental Figure 3

Supplemental Figure 4

Supplemental Figure 5

Supplemental Figure 6

Supplemental Figure 7

Related Manuscript File

Related Manuscript File

Related Manuscript File

Supplemental Information

## Data Availability

The RNAseq and iCLIP datasets were deposited in Gene Omnibus (GEO) with accession number GSE235358 and GSE235655. Any material or additional information required to replicate or reanalyze the data reported in this paper is available from the lead contact upon request.
