# Supplementary figures and images for: The RNA binding proteins TIA1 and TIAL1 promote Mcl1 mRNA translation to protect germinal center responses from apoptosis
Source: Cell Mol Immunol. 2023 Jul 20;20(9):1063–76. doi: 10.1038/s41423-023-01063-4 (PMC10469172; doi:10.1038/s41423-023-01063-4)

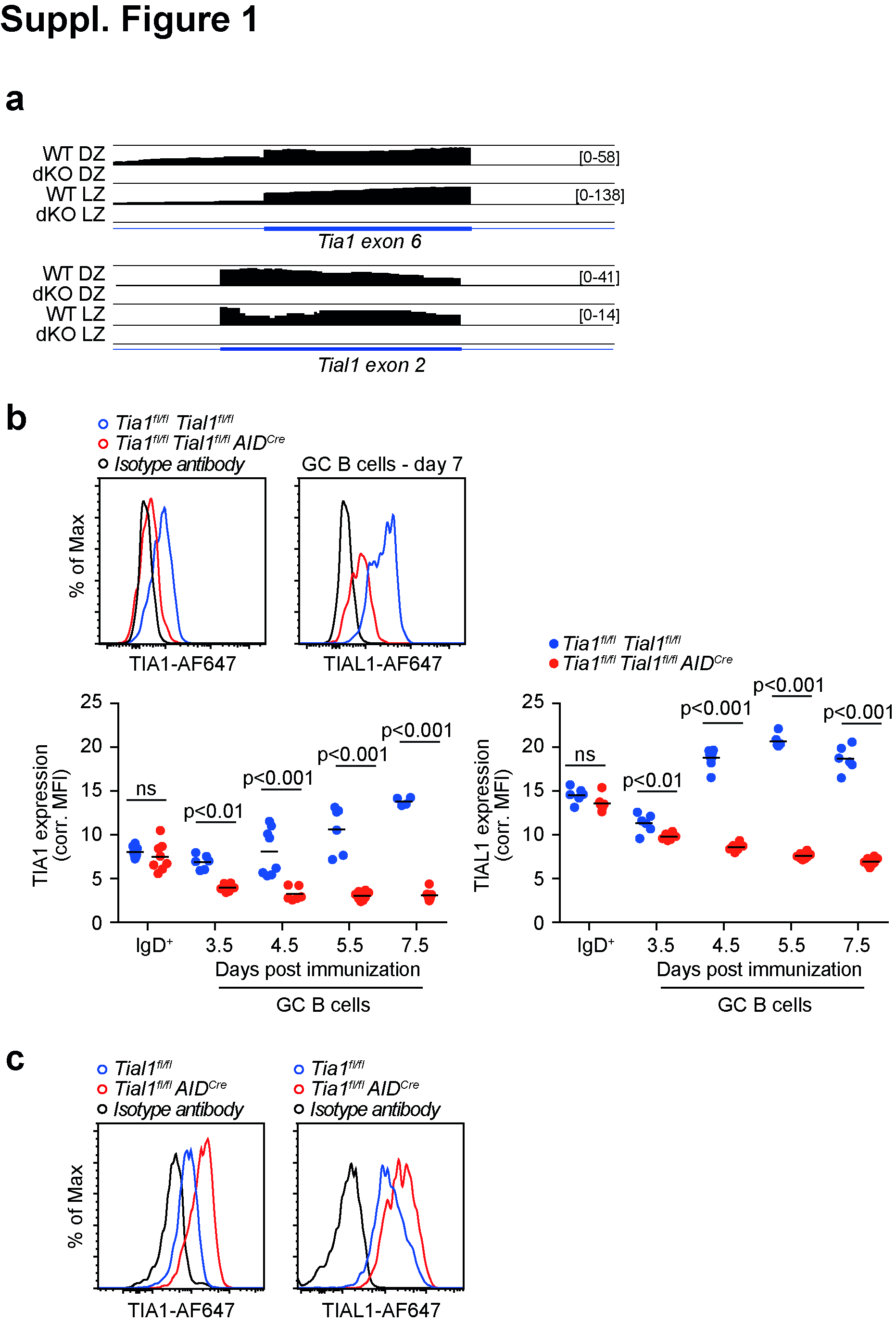

Supplement: Supplementary file 1 — Supplemental Figure 1 [file 41423_2023_1063_MOESM1_ESM.tif]

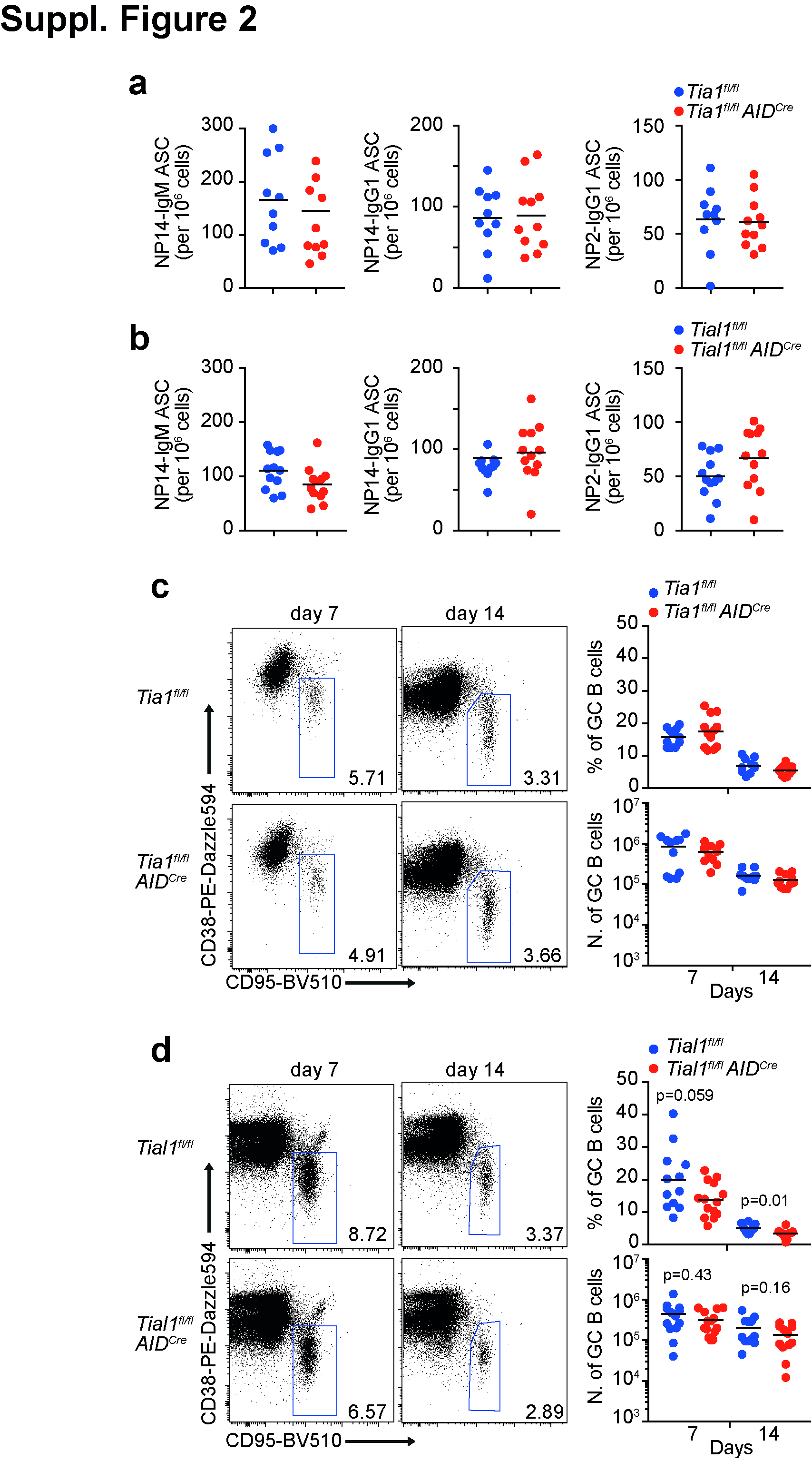

Supplement: Supplementary file 2 — Supplemental Figure 2 [file 41423_2023_1063_MOESM2_ESM.tif]

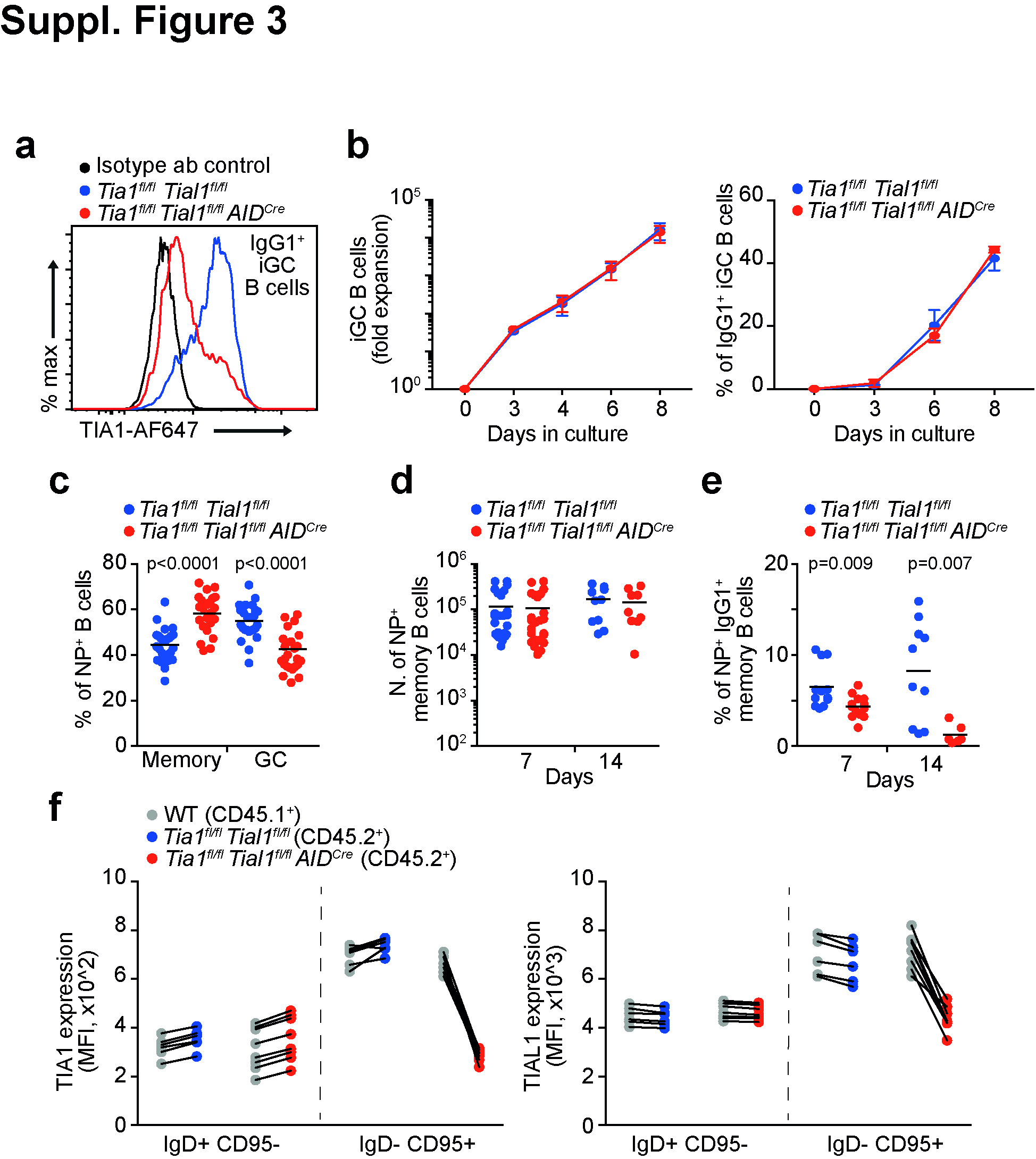

Supplement: Supplementary file 3 — Supplemental Figure 3 [file 41423_2023_1063_MOESM3_ESM.tif]

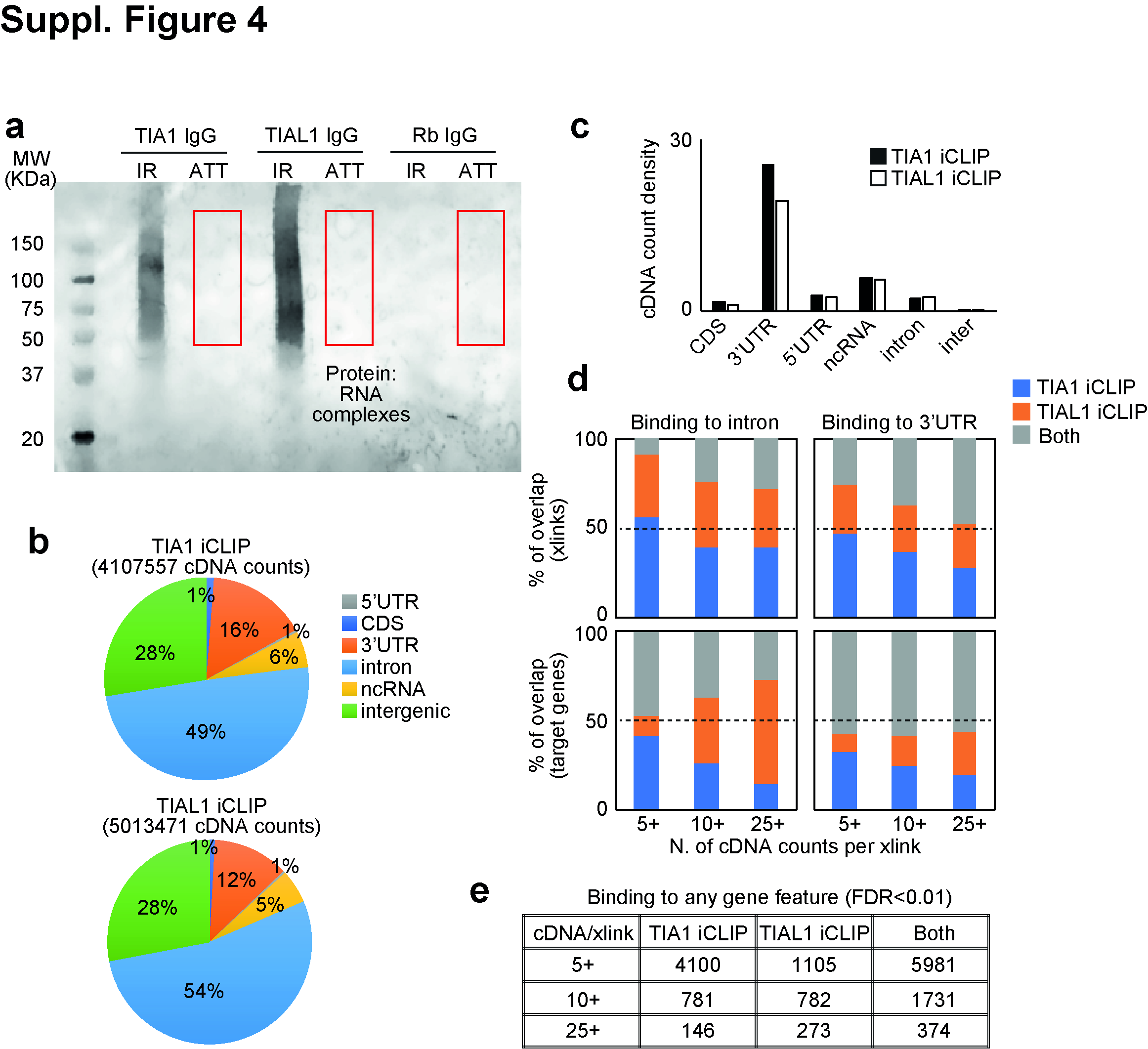

Supplement: Supplementary file 4 — Supplemental Figure 4 [file 41423_2023_1063_MOESM4_ESM.tif]

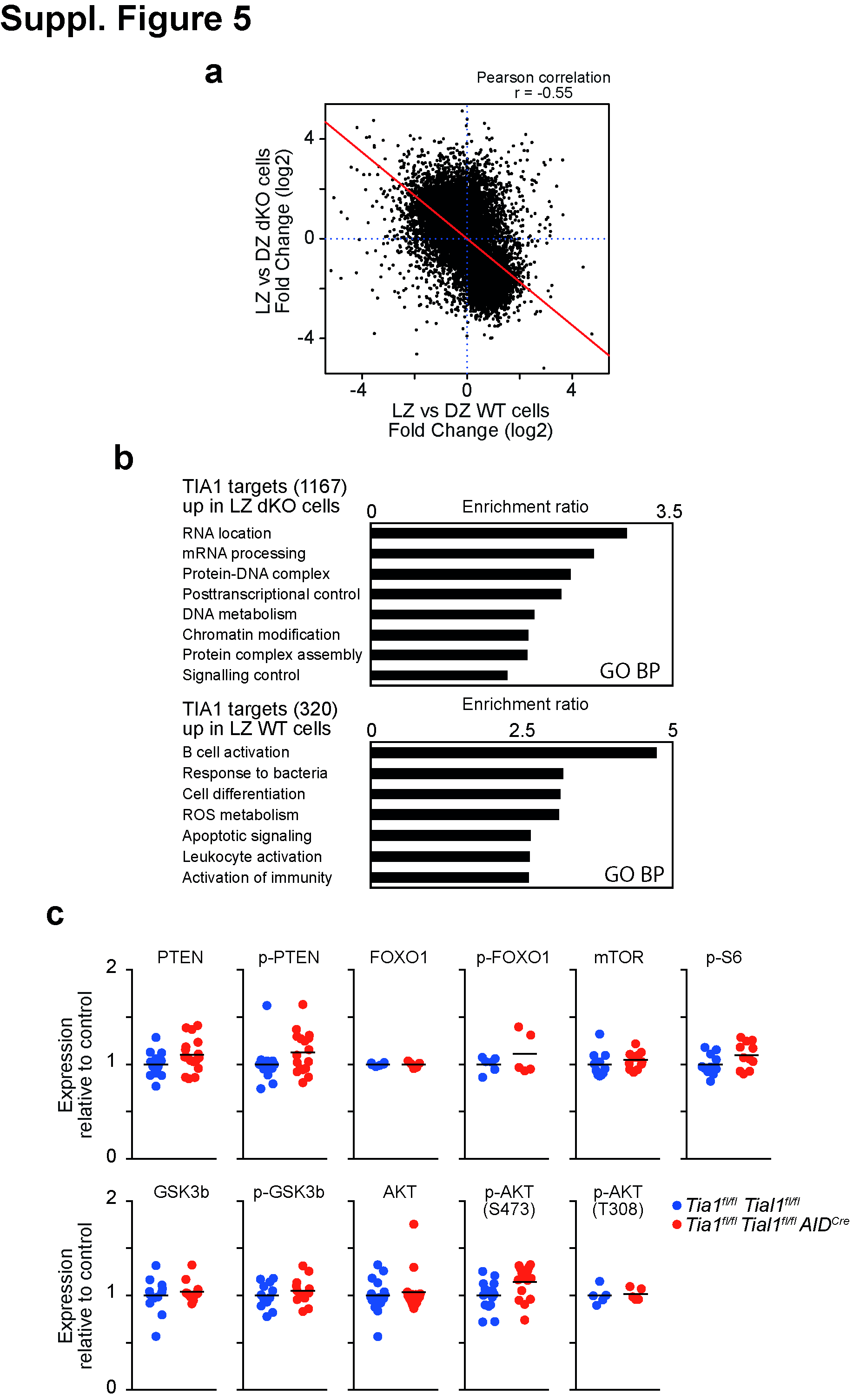

Supplement: Supplementary file 5 — Supplemental Figure 5 [file 41423_2023_1063_MOESM5_ESM.tif]

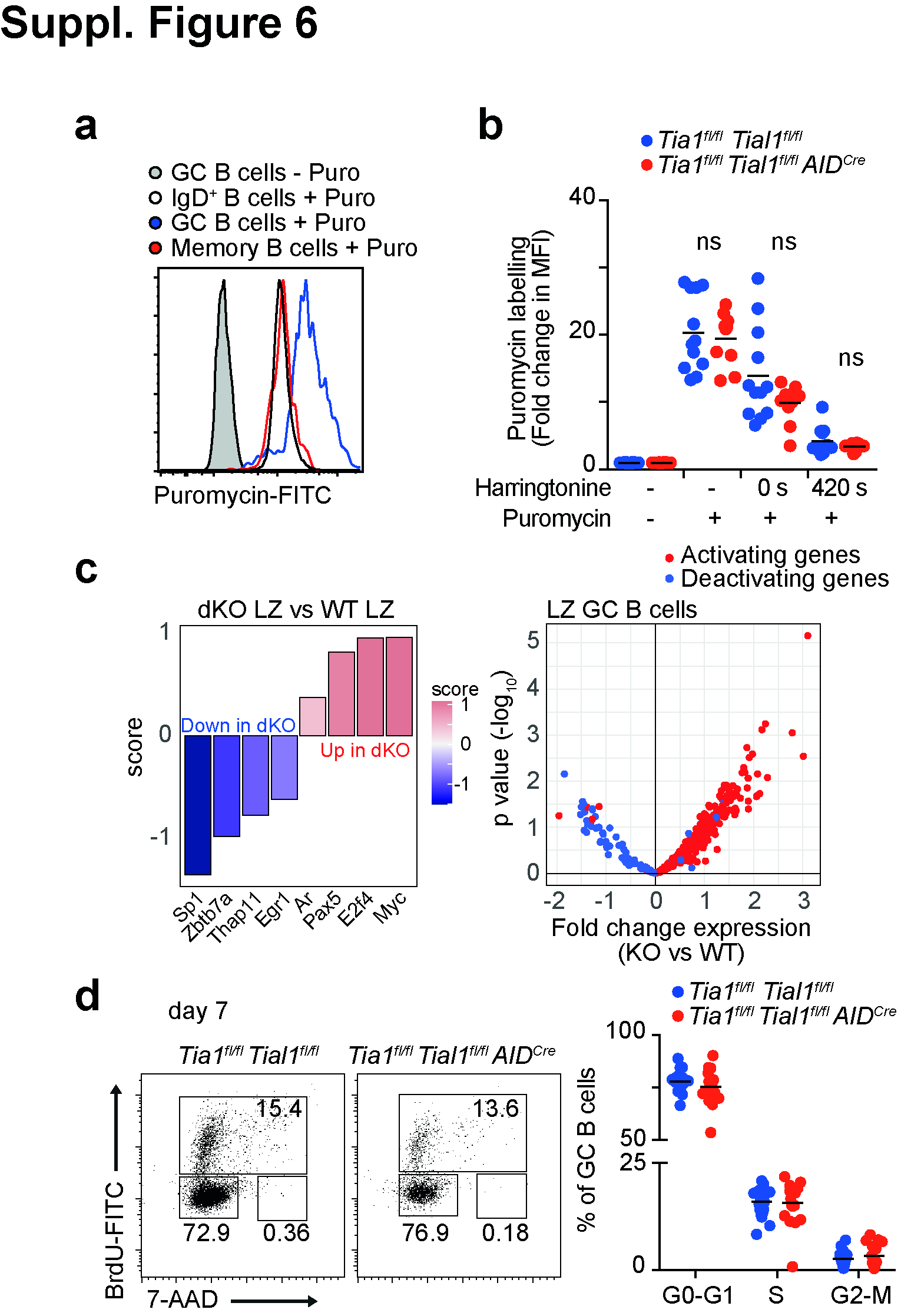

Supplement: Supplementary file 6 — Supplemental Figure 6 [file 41423_2023_1063_MOESM6_ESM.tif]

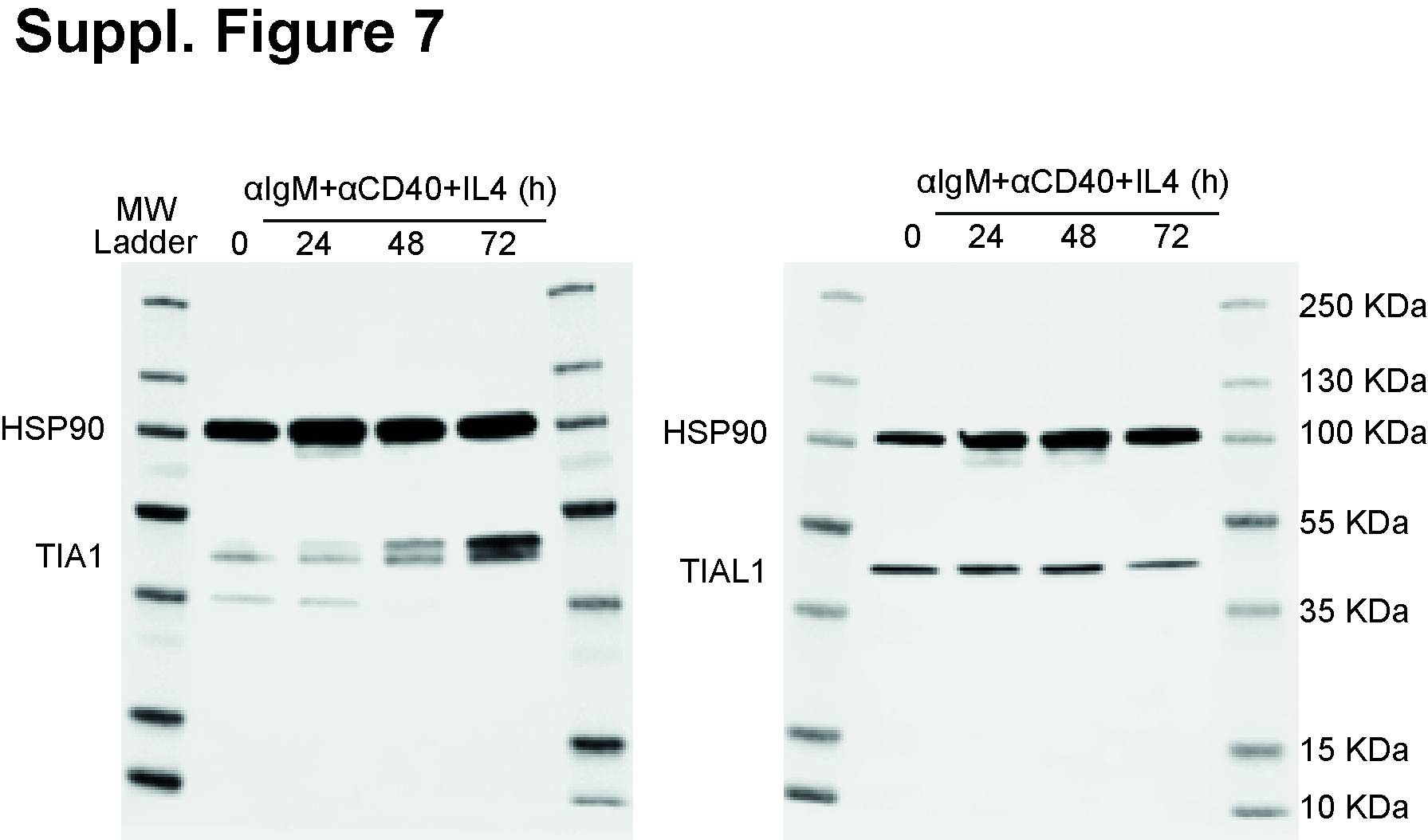

Supplement: Supplementary file 7 — Supplemental Figure 7 [file 41423_2023_1063_MOESM7_ESM.tif]
