# Supplementary material for: The RNA binding proteins TIA1 and TIAL1 promote Mcl1 mRNA translation to protect germinal center responses from apoptosis
Source: Cell Mol Immunol. 2023 Jul 20;20(9):1063–76. doi: 10.1038/s41423-023-01063-4 (PMC10469172; doi:10.1038/s41423-023-01063-4)
Supplement: Supplementary file 11 — Supplemental Information [file 41423_2023_1063_MOESM11_ESM.docx]

**SUPPLEMENTAL INFORMATION**

**Supplemental Figure 1. Single deletion of TIA1 or TIAL1 does not impair GC responses.**

**a,** Annotation of RNAseq reads to *Tia1* exon 6 and *Tial1* exon 2 in samples from control and *Tia1 Tial1* dKO DZ and LZ B cells. RNAseq from main Figure 5. **b,** Deletion of TIA1 and TIAL1 in GC B cells from *Tia1^fl/fl^ Tial1^fl/fl^ AID^Cre^* mice. Top panels, representative FACS histograms showing TIA1 or TIAL1 expression in GC B cells from the spleen at Day 7 post-immunization (ip). Bottom panels, quantitation of TIA1 and TIAL expression in GC B cells from LNs of immunized mice. MFI was corrected by the background signal of the isotype antibody control. Data are representative of one of the two independent experiments performed with n>6 mice per genotype. Mann‒Whitney t test analyses. **c,** Flow cytometry analysis of TIA1 and TIAL1 expression in GC B cells from *Tia1^fl/fl^ ^fl^ AID^Cre^* and *Tial1^fl/fl^ AID^Cre^* mice.

**Supplemental Figure 2. Single deletion of TIA1 or TIAL1 does not impair GC responses.**

**a, b,** Number of NP14-IgM-, NP14-IgG1- and high-affinity NP2-IgG1-specific antibody-secreting cells (ASCs) in the spleens of control, *Tia1^fl/fl^ AID^Cre^* and *Tial1^fl/fl^ AID^Cre^* mice at Day 14 after immunization. Data are from two independent experiments performed with n=4-6 mice per genotype. **c, d,** Quantitation of GC responses in the spleens of control*,* *Tia1^fl/fl^ AID^Cre^* and *Tial1^fl/fl^ AID^Cre^* mice at 7 and 14 days post-immunization with NP-KLH in alum. Left panels, representative dot plots. Right panels, quantitation of the percentage and number of GC B cells in two independent experiments performed each with at least n=4 mice per genotype. Mann‒Whitney tests were performed in statistical analyses.

**Supplemental Figure 3. Effect of TIA1 and TIAL1 deletion in IgG1^+^ and memory B cells.**

**a,** TIA1 deletion in in vitro-derived *Tia1^fl/fl^ Tial1^fl/fl^ AID^Cre^* GC-like B cells cultured for 6 days in the presence of IL-4. **b,** Quantitation of iGC B-cell expansion and the percentage of IgG1-class switched B cells. Data presented as the mean±SD are from one of the two independent experiments performed. Each experiment was performed with cells from two mice cultured independently. **c,** Percentage of NP-specific GC and memory B cells in control and *Tia1^fl/fl^ Tial1^fl/fl^ AID^Cre^* mice at Day 7 after immunization with NP-KLH. **d, e,** Quantitation of the total number of NP^+^ or NP^+^ IgG1^+^ memory B cells at Day 7 and Day 14 post-immunization. Data in c, d and e are from 2-3 independent experiments performed each with a minimum of n=5 mice per genotype. Mann‒Whitney tests. **d,** Assessment of TIA1 and TIAL1 deletion in CD45.2^+^ GC B cells in the bone marrow chimeras shown in Figure 4e.

**Supplemental Figure 4. Comparison of the RNA interactome of TIA1 and TIAL1 in B cells.**

**a,** Visualization of protein‒RNA complexes isolated for characterization of the RNA interactome of TIA1 and TIAL1 in B cells activated in vitro with αIgM, αCD40 and IL4 for 72 h. **b,** Pie charts showing the distribution of unique cDNA counts annotated to different genomic features in TIA1 and TIAL1 iCLIP assays. **c,** Analysis of the density of cDNA count annotated per crosslink site in TIA1 and TIAL1 iCLIP. **d,** Measurement of crosslink and gene target overlap in TIA1 and TIAL1 iCLIPs. Analysis is subdivided based on whether protein binds within introns or 3'UTRs. **e,** Quantitation of the number of genes identified as targets in TIA1 and TIAL1 iCLIP assays.

**Supplemental Figure 5. Extensive changes in the transcriptome of DZ and LZ B cells in the absence of TIA1 and TIAL1.**

**a,** Analysis of fold changes in gene expression in LZ versus DZ B cells in control and *Tia1^fl/fl^ Tial1^fl/fl^ AID^Cre^* mice. **b,** Gene Ontology enrichment analysis showing the top pathways associated with the TIA1 and TIAL1 gene targets upregulated in control or *Tia1* *Tial1* dKO LZ B cells (320 and 1167 genes, respectively). **c,** Quantitation of components of the PI3K pathway in GC B cells from control and *Tia1^fl/fl^ Tial1^fl/fl^ AID^Cre^* mice. The expression of the indicated proteins was measured by flow cytometry and relative quantified to the expression in control cells. Data are from 2 to 4 independent experiments with at least n=3 mice per genotype.

**Supplemental Figure 6. RNA translation and the cell cycle are not altered in *Tia1^fl/fl^ Tial1^fl/fl^ AID^Cre^* mice.**

**a,** Representative histogram of nascent peptide labeling with puromycin. **b,** Quantitation of puromycin labeling of new peptides in GC B cells from control and *Tia1^fl/fl^ Tial1^fl/fl^ AID^Cre^* mice. Cells were incubated with or without the translational initiation inhibitor harringtonine at the indicated times prior to the addition of puromycin for 10 min. Data from GC B cells of individual mice (n=12) are shown as MFI corrected by the background signal from an isotype control antibody. Data are representative of one of the two independent experiments performed. **c,** Analysis of transcription factor (TF) activity changes in LZ GC B cells from control and *Tia1^fl/fl^ Tial1^fl/fl^ AID^Cre^* mice. TF activities were inferred with the decoupleR package using transcriptomics data. Right panel, changes in the fold mRNA expression of activating and deactivating MYC-dependent genes. **d,** Analysis of cell cycle progression of GC B cells from control and *Tia1^fl/fl^ Tial1^fl/fl^ AID^Cre^* mice at Day 7 after immunization with NP-KLH. Left panels, pseudocolor dot plots showing representative FACS staining for analysis of short-term labeling of DNA with BrdU. Right panel, quantitation of the percentage at G_0-1_, S and G_2_-M phases of the cell cycle. Data are representative of one of the three independent experiments performed with at least 7 mice per group.

**Supplemental Figure 7. Unprocessed immunoblots.**

(Relative to main Figure 1e). Unprocessed and uncropped immunoblots showing TIA1, TIAL1 and HSP90 expression in B cells after treatment with anti-IgM, anti-CD40 and rmIL4

**Supplemental Table 1. RNA interactome analyses with iCLIP.**

**Supplemental Table 2. Transcriptome analyses with DESeq2.**

**Supplemental Table 3. List of antibodies.**
